# Multi-stage insecticide potentials of *Myristica fragrans* against the invasive *Aedes albopictus*, and putative binding mechanism of myristicin targeting the odorant-binding proteins (OBPs)

**DOI:** 10.1016/j.crpvbd.2026.100424

**Published:** 2026-08-07

**Authors:** Penny Humaidah Hamid, Arif Nur Muhammad Ansori, Mochammad Aqilah Herdiansyah, Herjuno Ari Nugroho, Ahmad Ghiffari, Mujiyanto Mujiyanto, Kartika Dyah Palupi

**Affiliations:** a Faculty of Animal Science, Universitas Sebelas Maret, Indonesia; b Genomic Department, Tropical Onehealth and Ecohealth Institute, Indonesia; c Institute of Research and Community Service, Universitas Sebelas Maret, Indonesia; d Postgraduate School, Universitas Airlangga, Indonesia; e Uttaranchal Institute of Pharmaceutical Sciences, Uttaranchal University, India; f Medical Biotechnology Research Group, Virtual Research Center for Bioinformatics and Biotechnology, Indonesia; g Department of Biology, Faculty of Science and Technology, Universitas Airlangga, Indonesia; h Research Center for Applied Microbiology, National Agency of Research and Innovation, (BRIN), Indonesia; i Department of Environment, Faculty of Medicine, Universitas Muhammadiyah Palembang, Indonesia; j Vectors and Vector-Borne Diseases Control, National Agency of Research and Innovation, (BRIN), Indonesia; k Research Center for Pharmaceutical Ingredients and Traditional Medicine, National Agency of Research and Innovation (BRIN), Indonesia

**Keywords:** Mosquito, Zoonotic, Vector, Olfactory, Deterrence, Insecticide

## Abstract

The Asian tiger mosquito, *Aedes albopictus*, is native to Southeast Asia and an invasive species with rapid geographical expansion. *Aedes albopictus* has contributed to the spread of numerous infectious diseases affecting both humans and animals. This study evaluated the insecticidal potential of *Myristica fragrans* essential oil (MFEO) from Maluku Island against multiple life stages of *Ae. albopictus*. MFEO completely inhibited egg hatching from 0.001% (w/v), with treated eggs remaining unhatched throughout a 25-day observation period; scanning electron microscopy revealed structural damage to the exochorionic layer, indicating that disruption of the egg surface impairs embryonic development. In larvicidal assays, MFEO showed rapid, concentration- and time-dependent toxicity, achieving 100% mortality within 30 min at concentrations of 100 ppm and above, comparable to temephos; at 1–50 ppm, complete lethality was reached within 120 min. Against adults, MFEO produced a 30-min knockdown concentration (KD_50_) of 1.545% and a 24-h lethal concentration (LC_50_) of 0.270%. MFEO-treated ovitraps received no eggs, whereas controls attracted 78–90 eggs in 48 h. Gas chromatography-mass spectrometry identified myristicin (35.35%) as the principal constituent, with α-terpineol comprising 10.74% and phenol, 2,4,6-tris(1-phenylethyl)- comprising 11.24%. Molecular docking against 19 annotated odorant-binding proteins (OBPs) of *Ae. albopictus* showed the strongest binding of myristicin to OBP3 (−6.8 kcal/mol), mediated by van der Waals, hydrophobic and polar hydrogen interactions; molecular dynamics validation (CABS-flex) of the myristicin-OBP3 complex yielded a mean root-mean-square fluctuation of 1.468 Å, i.e. below the 2 Å stability threshold. Further docking against acetylcholinesterase (AChE), GABA receptors and voltage-gated sodium channels (VGSC) supported a neurotoxic component. Together, these results indicate that MFEO disrupts multiple life stages through combined chemosensory interference, eggshell damage and neurotoxic action. The multi-target, multi-stage activity observed in MFEO, together with its behavioral deterrent effects, suggests it warrants further investigation as a botanical candidate for integrated vector management, potentially offering an alternative to some conventional neurotoxic insecticides. Further, functional validation by bio-guided fractionation and target-specific assays is recommended.

## Highlights

• Essential oil of *Myristica fragrans* from Banda Neira, Indonesia, targets multiple mosquito life stages.
• Complete egg hatch inhibition at ≥ 0.001% with SEM evidence of exochorionic damage.
• Multi-stage efficacy positions MFEO as a promising integrated vector management tool.
• Myristicin-OBP-3 docking revealed a potential mechanism for oviposition deterrence.
• Disruption of OBP-mediated olfaction may explain behavioural deterrence.

## Graphical abstract

**Figure:**
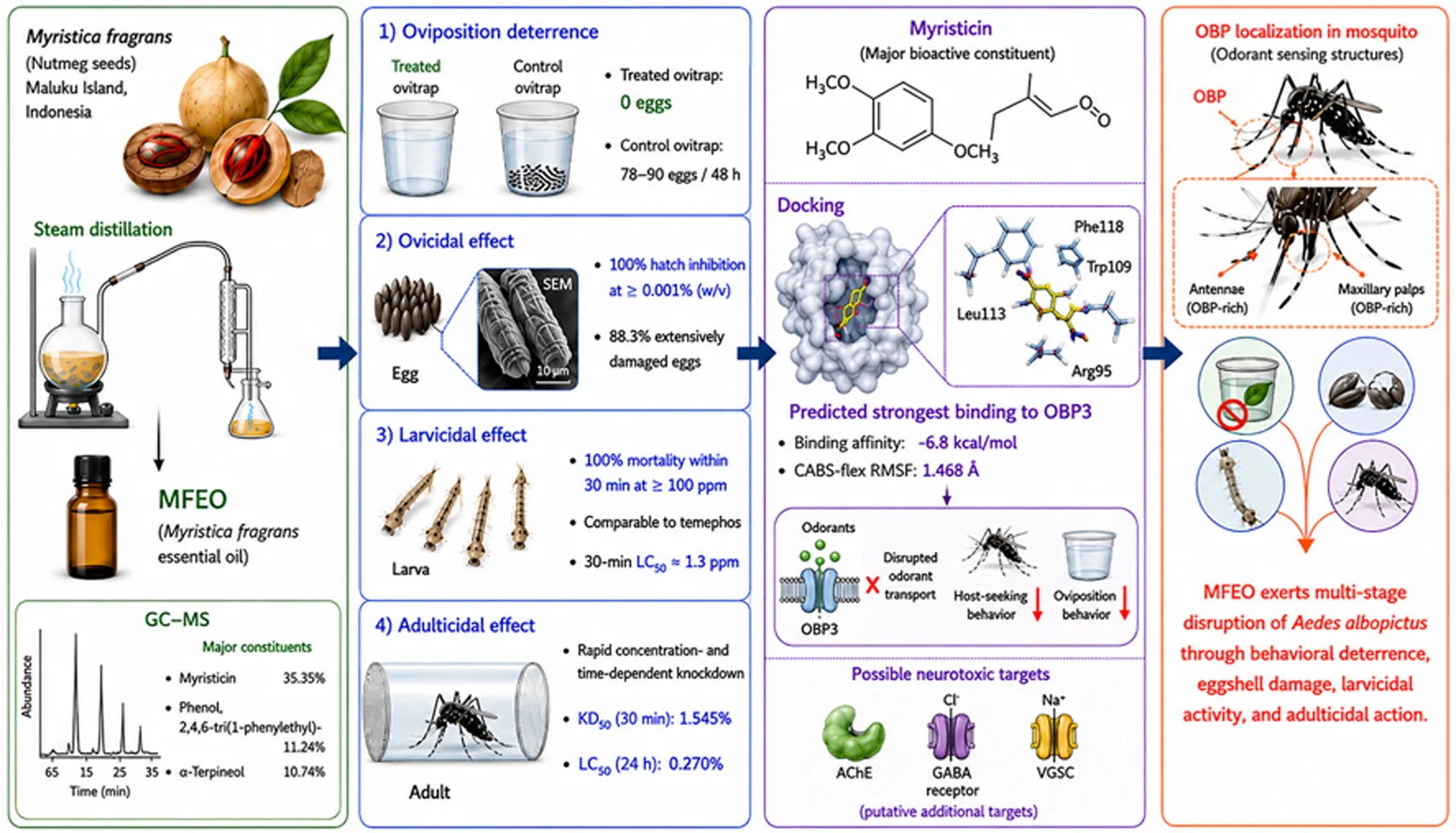

## 1 Introduction

The Asian tiger mosquito, *Aedes albopictus*, is a globally invasive species, posing a substantial threat to public health. This species plays a role as a vector of arboviruses such as dengue, chikungunya, and Zika (Benedict et al., 2007; Bonizzoni et al., 2013; Kraemer et al., 2015; Gloria-Soria et al., 2020). The global spread of *Ae. albopictus* is largely driven by the transport of drought-resistant eggs, which allows the species to survive prolonged travel and establish in new geographical regions (Kraemer et al., 2015, 2019). Further, the population establishment is facilitated by its adaptability to diverse climatic conditions and preference for breeding in human-modified environments, and has exacerbated vector control challenges (Kamal et al., 2018; Reinhold et al., 2018). The opportunistic feeding behaviour on a wide range of hosts, from cold-to warm-blooded animals, underscores the potential of *Ae. albopictus* to participate in diverse vertebrate-virus transmission cycles. Notably, *Ae. albopictus* exhibits a strong preference for human hosts when given a choice among vertebrates such as calves, chickens, dogs, and goats, further confirming its high degree of anthropophily (Delatte et al., 2010). This behaviour, combined with its propensity for multiple blood-feeding events in mixed host environments, significantly increases the risk of arbovirus transmission to human populations through interrupted feeding patterns. Anthropogenic changes, such as urbanisation, are known to influence mosquito communities by altering ecological dynamics at the interface of natural and human environments, thereby modulating the spill-over risks of zoonotic pathogens (Pereira-Dos-Santos et al., 2020). Despite the widespread reliance on conventional chemical insecticides for control, the emergence of insecticide-resistant mosquito populations, coupled with growing concerns over their environmental and human health impacts, underscores the pressing need for sustainable and effective alternatives to manage this invasive vector (Reinhold et al., 2018).

Essential oils have gained attention as eco-friendly, biodegradable substitutes for synthetic insecticides, with several plant oils demonstrating effective repellent activity against blood-feeding arthropods (Park and Tak, 2016; Spinozzi et al., 2021). However, limited research exists on the efficacy of *Myristica fragrans* Houtt. essential oil (MFEO) against specifically *Ae. albopictus* in multiple life cycle stages. Native to the Indonesian Maluku Islands, *M. fragrans* has long been valued for its culinary and medicinal applications (Van Gils and Cox, 1994; Brixius, 2018). The seed comprises a red aril (mace) and a brown inner kernel (nutmeg), both rich in bioactive compounds such as myristicin, eugenol, and safrole, which have shown insecticidal, repellent, and growth-regulating effects across various insect species (Park and Tak, 2016). *Myristica fragrans* is well-known to possess antioxidant, antimicrobial, anti-inflammatory, and other therapeutic properties, alongside notable pest control effects (Abourashed and El-Alfy, 2016; Ashokkumar et al., 2022). For example, MFEO exhibited antifeedant activity in *Lymantria dispar* larvae and insecticidal activity against *Blattella germanica*, *Chrysomya albiceps* and *Musca domestica* (Jung et al., 2007; Cossetin et al., 2021).

To date, most botanical insecticide studies have examined isolated life stages of mosquitoes, typically focusing on either larval or adult mortality. Integrated assessments that simultaneously evaluate oviposition deterrence, ovicidal activity, larvicidal efficacy, and adulticidal effects within a single experimental framework remain limited. In this study, we investigated the insecticidal efficacy of MFEO against *Ae. albopictus* across multiple developmental stages, with particular emphasis on oviposition deterrence, ovicidal activity, larvicidal potency, and adult knockdown effects. While the observed bioactivity across life stages is promising, limited information is available regarding the molecular mechanisms underlying these behavioural disruptions, especially those associated with mosquito olfaction. Odorant-binding proteins (OBPs) are central components of the mosquito olfactory system. They function as molecular shuttles that transport hydrophobic odorant molecules through the sensillar lymph to olfactory receptors, thereby initiating behavioural responses involved in host-seeking, feeding, and oviposition (Deng et al., 2013; Wheelwright et al., 2021). Disruption of OBP-ligand interactions may impair odor perception, compromise host detection, and alter oviposition site selection. To complement the multi-stage bioassays, we performed *in silico* molecular docking analyses to evaluate the binding interactions between myristicin, the major constituent of MFEO, and 22 OBPs identified in *Ae. albopictus*. This integrative approach provides mechanistic insight into potential molecular targets of MFEO and strengthens the evidence for its targeted behavioural disruption, supporting its development for mosquito control.

## 2 Materials and methods

### 2.1 *Myristica fragrans*

*Myristica fragrans* collections were conducted under national guidelines for commercially valued goods (MoARI, 2021); collections complied with all local and national guidelines. The seeds were collected under the supervision and permission issued to the authors for *M. fragrans* garden in Banda Neira, Central Maluku Regency, Maluku Province (Supplementary Fig. S1), which is producing essential oil for commercial purposes. In this study, only the dried nut (kernel/endosperm) was subjected to steam distillation for essential oil extraction. The yellow pericarp and the red aril (mace) were not included in the distillation process. Ten kg of *M. fragrans* seeds were first dried at 50 °C for 8 h in a drying cabinet, followed by steam distillation at 100 °C for 4–5 h using a Clevenger-type apparatus. This extraction process yielded 400 ml of pure essential oil with a light-yellow colour, which was subsequently stored in a sterile glass container.

### 2.2 Mosquitoes

*Aedes albopictus* eggs were collected from Boyolali, Central Java (7°22′23.5″S, 110°43′59.8″E) using glass ovitraps externally coated with black stain, as described in our previous studies on *Aedes aegypti* (Hamid et al., 2018, 2020; Martianasari and Hamid, 2019). Each ovitrap was fitted with a filter paper at the opening to facilitate egg deposition and was replenished weekly with fresh water and filter paper throughout the collection period from January to March 2023. Collection sites were randomly selected, prioritizing cattle pen communities surrounded by dense vegetation to closely mimic natural breeding habitats. Following collection, egg-bearing filter papers were air-dried at ambient room temperature and stored in plastic containers for preservation. Larvae were subsequently induced to hatch and were reared on a diet of chicken liver, provided in both wet and dried forms, until reaching the pupal stage. Emerged adult *Ae. albopictus* were selected and maintained on a 10% sucrose solution absorbed in cotton balls for sustenance. Mosquito condition and rearing protocols were adopted from our previous study on *Ae. aegypti* (Martianasari and Hamid, 2019). To ensure consistency across experimental procedures, adult *Ae. albopictus* from the field-collected cohort (generation F_0_) were selected for controlled laboratory mating to establish successive filial generations. Artificial feeding sourced from human blood (Palang Merah Indonesia, Boyolali, Indonesia) was modified and provided to adults to support egg production. This approach successfully yielded generations F_1_ to F_3_, which were reared under standardized laboratory conditions.

### 2.3 Egg hatchability evaluation

Ovicidal activity of MFEO was evaluated against freshly collected *Ae. albopictus* eggs. Whatman No. 1 filter papers containing newly laid eggs were exposed to MFEO solutions prepared in ethanol and diluted to final concentrations of 1%, 0.1%, 0.01%, and 0.001% (w/v). The final ethanol concentration in all treatments did not exceed 1% (v/v). For each concentration, approximately 50 eggs were used per replicate, and assays were conducted in triplicate (total *n* = 150 eggs per concentration). Treated filter papers were placed in a BOD incubator (Memmert GmbH, Boyolali, Germany) under controlled laboratory conditions (25 ± 2 °C, 70–80% relative humidity, RH). Eggs remained in continuous contact with treated substrates throughout the exposure period. Eggs were inspected daily, and any newly emerged larvae were counted and immediately removed from the treated substrate; this daily removal also prevented cannibalism between newly emerged and more developed larvae, which could otherwise lead to an underestimation of hatchability. Hatchability was calculated based on the cumulative number of larvae removed over the 25-day observation period. Hatch inhibition (%) was calculated relative to the control group.

### 2.4 Scanning electron microscopy

Freshly oviposited *Ae. albopictus* eggs (0–24 h old) were obtained. Batches of 50 eggs were immersed for 60 s in either MFEO solution at the 24-h LC_50_ (0.27% v/v in 10% ethanol) or the solvent control (10% ethanol). Treated eggs were air-dried, held at 27 ± 1 °C and 80 ± 5% RH, and after 24 h were processed for SEM. Eggs were fixed in 2.5% glutaraldehyde in 0.1 M phosphate buffer (pH 7.2) for 4 h, post-fixed in 1% OsO_4_ for 1 h, dehydrated through a graded ethanol series (30–100%), critical-point dried, mounted on aluminium stubs, and sputter-coated with gold-palladium. Imaging was carried out with a JSM-IT200 SEM (JEOL, Tokyo, Japan) at 10 kV at the Genomic Laboratory, BRIN, Indonesia.

Thirty eggs per treatment were randomly selected from separate stubs and imaged at 1000× and 5000× (central and polar regions). Two independent observers blinded to treatment scored each egg for the percentage of the imaged exochorionic surface displaying: (i) loss of tubercle integrity (fragmented, collapsed, or absent); (ii) disruption of the reticulate meshwork; and (iii) occluded or malformed aeropyles. An egg was classified as “extensively damaged” if > 50% of its surface exhibited clear structural alteration. Inter-observer agreement exceeded 90%; discordant cases were re-evaluated and resolved by consensus. Where intact tubercles could be identified, approximate height and basal diameter were measured using ImageJ on 5 randomly chosen tubercles per egg (150 tubercles per treatment). A Mann-Whitney test was used to compare tubercle dimensions between control and treated eggs, with significance set at *P* < 0.05.

### 2.5 Larval mortality test

Larvicidal activity of MFEO was evaluated against third-instar *Ae. albopictus* larvae following a modified WHO bioassay protocol. MFEO stock solution was prepared in ethanol and serially diluted with distilled water to obtain final test concentrations of 0.1%, 0.05%, 0.01%, 0.005%, 0.001%, and 0.0001% (w/v). These correspond to 1000, 500, 100, 50, 10, and 1 ppm, respectively. Temephos (Sigma-Aldrich, Buchs, Switzerland) was prepared at the same nominal concentrations for comparative purposes. For each concentration, 60 larvae were placed in 25 ml of test solution within a 100-ml cup, and each treatment was performed in triplicate. A solvent control containing ethanol at the same final concentration as the treated groups (not exceeding 1% v/v) was included. Assays were conducted at 25 ± 2 °C under controlled laboratory conditions without larval feeding during exposure.

Larval mortality was recorded at 30, 60, 90, and 120 min, and at 24 h. Larvae were considered dead if they showed no movement upon gentle probing with a fine brush. Dead larvae were removed at each observation time to prevent decomposition products from affecting surviving larvae. Mortality data were corrected using Abbot’s formula when necessary. Because complete mortality (100%) was achieved at all concentrations by 120 min, probit regression was applied to the 30-min mortality data to estimate LC_50_ with 95% confidence intervals.

### 2.6 Adulticidal bioassay

The WHO tube test (WHO, 2016) was used. Permethrin (Sigma-Aldrich, Steinheim am Albuch, Germany) and MFEO were each prepared at six concentrations (10%, 1%, 0.1%, 0.01%, 0.001%, and 0.0001% (w/v)). Permethrin was dissolved in water, while MFEO was dissolved in ethanol. For each concentration, 2 ml of solution was applied evenly to Whatman No. 1 filter paper (12 × 15 cm), air-dried in the dark for 2 h, and placed inside a WHO holding tube. Control tubes received ethanol-only treated papers. Unfed female *Ae. albopictus* were introduced in batches of 20 per tube. Three replicates were performed for each concentration. Knockdown (inability to stand, walk, fly, or retain wing integrity) was recorded at 30, 60, 90, and 120 min. After 120 min, mosquitoes were transferred to clean holding tubes with 10% sugar water and kept for a further 24 h, at which point mortality/lethal concentration was scored.

The chosen range includes the WHO diagnostic dose for permethrin against *Ae. albopictus* (0.25–0.75%) and extends four orders of magnitude downward to capture the full dose-response curve. The lowest concentration (0.0001%) was expected to be well below the KD_50_ (knockdown) for all compounds, ensuring that the sigmoid curve would be anchored at the bottom. Early readings are reported as knockdown (KD_50_), while 24 h data are reported as lethal concentration (LC_50_), following the WHO terminology.

### 2.7 Oviposition deterrence bioassay

Oviposition deterrent activity of MFEO was evaluated using gravid *Ae. albopictus* females. For each replicate, 30 mated females (3–5 days post-blood-feeding) were introduced into a screened cage (30 × 30 × 30 cm). A 10% sucrose solution was provided *ad libitum* throughout the assay. Two ovitraps were placed diagonally within each cage: (i) a treatment ovitrap containing MFEO solution at concentrations of 1%, 0.1%, 0.01%, and 0.001% (w/v); and (ii) a control ovitrap containing tap water with 1% ethanol (solvent control). Each ovitrap contained 200 ml of liquid, with MFEO prepared from stock solution and thoroughly homogenized before use. Whatman No. 1 filter paper strips were positioned along the inner wall of each ovitrap to serve as oviposition substrates. Mosquitoes were allowed to oviposit for 48 h under controlled laboratory conditions (25 ± 2 °C, 70–80% RH, 12:12 h light:dark photoperiod). After exposure, filter papers were removed, air-dried, and the number of eggs deposited on each substrate was counted under a dissecting microscope (Euromex, Duiven, Netherlands). The oviposition deterrence index (ODI) was calculated as:

$$\text{ODI}=\frac{N_{c}-N_{t}}{N_{c}+N_{t}}$$

where $N_{c}$ is the number of eggs in the control ovitrap and $N_{t}$ is the number of eggs in the treated ovitrap. Each treatment was conducted in triplicate, and data are presented as the mean ± standard deviation.

### 2.8 Gas chromatography-mass spectrometry analysis of *Myristica fragrans* essential oil

Gas chromatography-mass spectrometry (GC-MS) analysis of MFEO was performed at the Advanced Characterization Laboratory of the BRIN, Indonesia. The essential oil was prepared by diluting it in dichloromethane to a final concentration of 1000 μg/ml, followed by filtration through a 0.22 μm Minisart syringe membrane to ensure sample purity. The analysis utilized a Shimadzu GCMS-QP2020NX system (Shimadzu, Kyoto, Japan), equipped with an Rtx-5MS column (30 m in length and 0.25 mm in diameter) composed of 5% diphenyl and 95% dimethylpolysiloxane. Ultra-high-purity helium served as the carrier gas, maintained at a pressure of 30 kPa. The injector was operated at 200 °C, the ion source at 230 °C, and the interface at 280 °C, with injections performed in splitless mode. The oven temperature program commenced at 60 °C, increased at a rate of 10 °C per min until reaching 150 °C, and was held at 150 °C for 3 min. Compound identification was conducted by comparing the obtained chromatographic profiles and mass-to-charge (m/z) ratios with the National Institute of Standards and Technology (NIST-17) database.

### 2.9 Molecular docking analysis

Preparation of molecular docking materials begins with retrieving test receptors according to Supplementary Tables S1–S3 on the UniProt web server (https://www.uniprot.org/). All test receptors were prepared by removing water molecules using PyMol v.1.74 software (Schrodinger LLC, New York, USA) to remove water molecules and purify the receptors. Furthermore, the test compound myristicin was retrieved from the PubChem web server (https://pubchem.ncbi.nlm.nih.gov/) with PubChem CID 4276. Molecular docking experiments were performed using PyRx 0.8 software (SourceForge Headquarters, San Diego, California, USA). Visualization of ligand-receptor complexes was performed using BIOVIA Discovery Studio 2019 software (Dassault Systèmes BIOVIA, San Diego, USA).

### 2.10 Validation of the molecular docking test

Validation of the molecular docking results was performed using the CABS-flex 3.0 web server (https://lcbio.pl/cabsflex3/), which enables efficient coarse-grained simulations of protein and peptide structural flexibility. The analysis was conducted to assess the dynamic behavior and stability of the receptor-ligand complex.

The myristicin-OBP3 complex, which exhibited the strongest binding affinity in the docking analysis, was selected for further simulation. CABS-flex parameters were set to include protein rigidity, distance restraints, C-alpha restraints weight, side-chain restraint weight, trajectory settings, temperature range, number of cycles, and RNG seed.

Structural fluctuations were evaluated based on Root Mean Square Fluctuation (RMSF) values derived from the simulation trajectories, which were used to characterize the stability of the protein-ligand complex.

### 2.11 Statistical analysis

Dose-response data for knockdown (KD, measured at 30, 60, 90, and 120 min) and mortality (LC, measured at 24 h) were analyzed by non-linear regression using a variable-slope, four-parameter log-logistic model. Knockdown/mortality percentages from three independent replicates were averaged at each concentration before fitting. Median knockdown concentrations (KD_50_) and median lethal concentrations (LC_50_) with their 95% confidence intervals were interpolated from the fitted curves. Goodness-of-fit was assessed by the coefficient of determination (*R*^2^) and, where applicable, by a lack-of-fit F-test (or Hosmer-Lemeshow test for probit models); all curves achieved *R*^2^ > 0.90 and non-significant lack-of-fit (*P* > 0.05), indicating adequate model performance. For adulticidal assays, the lowest tested concentration (0.0001%) yielded < 5% effect for all test substances, confirming that the concentration range was sufficient to anchor the lower plateau of the sigmoidal curve and that the estimated median values are robust. Differences between test substances were assessed by non-overlap of 95% confidence intervals or by relative median potency ratios where appropriate.

All statistical analyses and graphical representations were performed using GraphPad Prism version 8.02 (GraphPad Software, Boston, USA).

## 3 Results

### 3.1 Ovicidal efficacies

**Fig. 1:**
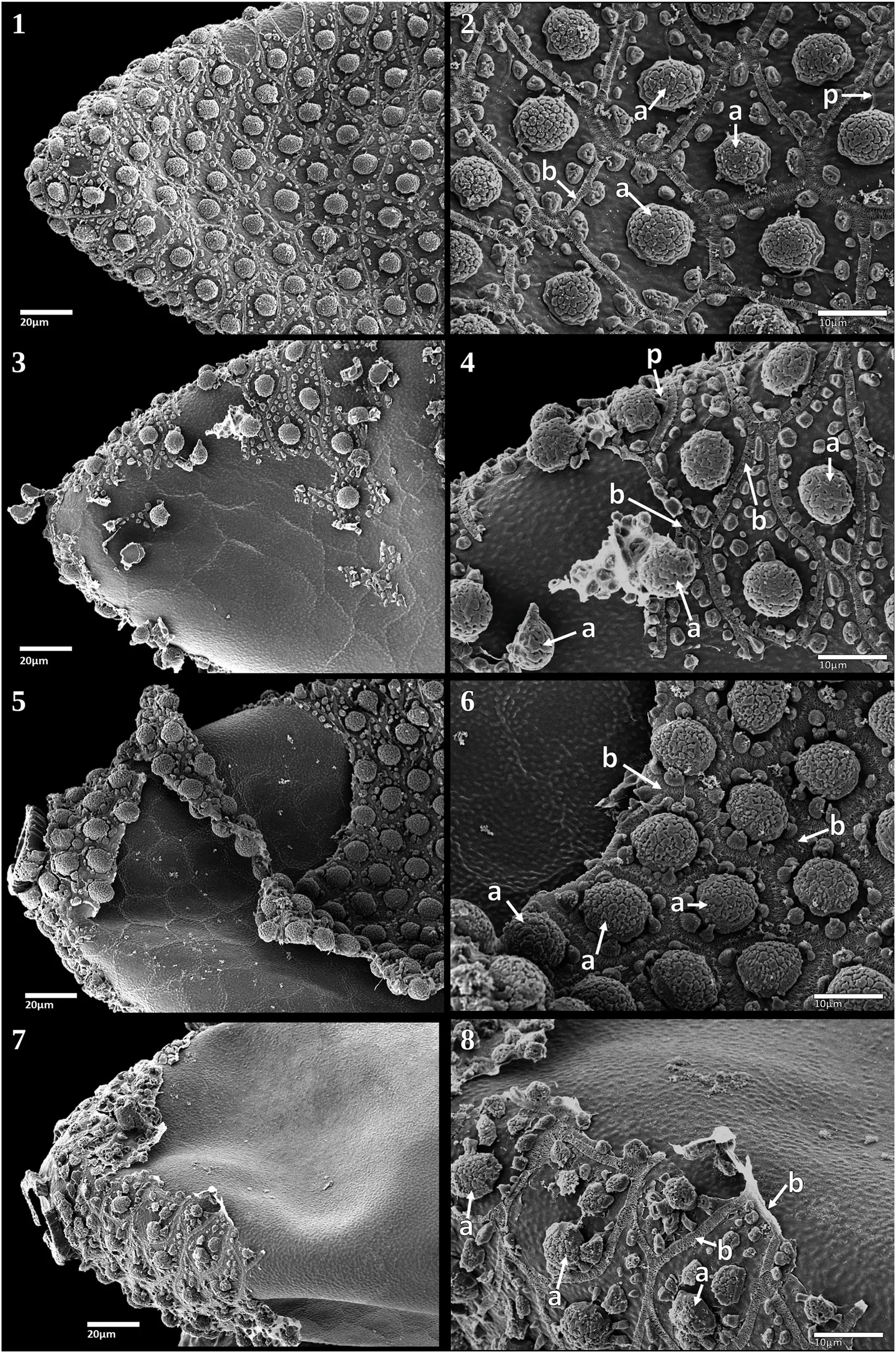
Scanning electron micrographs of *Aedes albopictus* eggs after exposure to *Myristica fragrans* essential oil (MFEO) at the 24 h LC_50_ (0.27% v/v in 10% ethanol) or to solvent control (10% ethanol). **1**, **2** Solvent-control eggs showing an intact exochorion with well-defined dome-shaped tubercles, a continuous reticulate meshwork, clear cellular boundaries, and patent aeropyles. **3**-**8** MFEO-treated eggs showing extensive structural disruption: tubercles are fragmented, collapsed, or dissolved; the reticulate meshwork is torn or absent; cell borders are irregular; and many aeropyles are occluded by amorphous debris or have lost their distinct rim structure.

Eggs exposed to MFEO remained unhatched throughout the 25-day monitoring period, whereas control eggs hatched normally. Scanning electron micrographs of untreated eggs (Fig. 1, panels 1, 2) showed an intact exochorion with well-defined dome-shaped tubercles, a continuous reticulate meshwork, clear cellular boundaries, and patent aeropyles. In contrast, MFEO-treated eggs (Fig. 1, panels 3–8) displayed extensive structural disruption. Tubercles were fragmented, collapsed, or entirely dissolved; the meshwork was torn or absent; cell borders were irregular; and many aeropyles appeared occluded with amorphous debris or had lost their distinct rim structure. Semi-quantitative scoring confirmed that 88.3% of treated eggs were extensively damaged (> 50% surface affected), whereas only 3.3% of control eggs showed comparable irregularity. Where residual tubercles could be measured, MFEO significantly reduced their height (mean ± SD: 0.42 ± 0.11 *vs* 0.82 ± 0.14 μm) and basal diameter (mean ± SD: 1.25 ± 0.28 *vs* 2.31 ± 0.35 μm) (*n* = 150 tubercles per treatment; Mann-Whitney U-test, *P* < 0.001).

### 3.2 Larvicidal assay

**Fig. 2:**
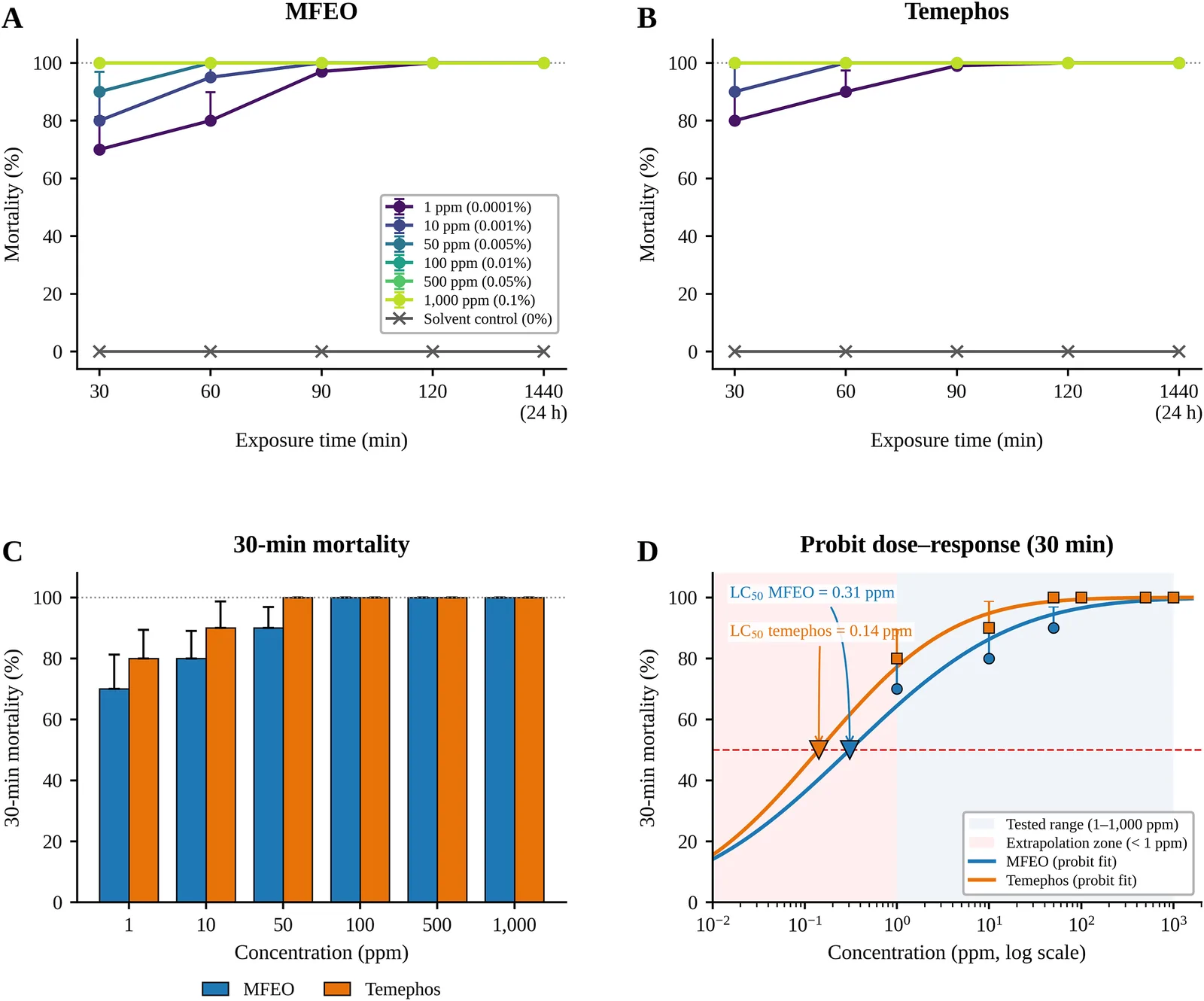
Larvicidal activity of *Myristica fragrans* essential oil (MFEO) compared with the synthetic organophosphate temephos against third-instar larvae of *Aedes albopictus*. **A**, **B** Time-course of mortality for MFEO and temephos, respectively, at each tested concentration. **C** 30-min mortality compared between MFEO (*blue*) and temephos (*orange*); this is the only time-point at which inter-concentration variation persisted, as 100% mortality was reached by 120 min at all concentrations and sustained through 24 h. **D** Probit-derived dose-response at 30 min, with fitted log-logistic curves overlaid on the empirical means; triangles mark the extrapolated LC_50_ values (MFEO 0.31 ppm, 95% CI: 0.15-0.63 ppm; temephos 0.14 ppm, 95% CI: 0.06-0.34 ppm). The blue band marks the tested concentration range; the red band marks the sub-1 ppm region in which the LC_50_ estimates lie. Because the lowest tested concentration (1 ppm) already produced ≥ 70% mortality at 30 min, the lower limb of the sigmoidal response was not empirically anchored; the LC_50_ values therefore fall outside the tested range and are reported as extrapolated estimates. Symbols represent mean ± SD of three independent replicates (*n* = 60 larvae per replicate). Solvent control (ethanol ≤ 1% v/v) showed 0% mortality at all time-points and is omitted for clarity. No delayed mortality or recovery was observed at 24 h.

MFEO demonstrated rapid, concentration- and time-dependent larvicidal activity against third-instar *Ae. albopictus* (Fig. 2, Table 1). At concentrations ≥ 100 ppm (≥ 0.01%), both MFEO and temephos caused 100% mortality within 30 min. At 50 ppm (0.005%), MFEO achieved 90% mortality at 30 min and reached 100% by 60 min, whereas temephos produced 100% mortality at 30 min. At 10 ppm (0.001%), MFEO produced 80% mortality at 30 min, increasing to 95% at 60 min and 100% by 90 min, whereas temephos showed 90% mortality at 30 min and reached 100% by 60 min. At the lowest concentration tested, 1 ppm (0.0001%), MFEO produced 70% mortality at 30 min, progressing to 80% at 60 min, 97% at 90 min, and 100% by 120 min; temephos produced 80% mortality at 30 min, 90% at 60 min, 99% at 90 min, and 100% by 120 min. No mortality was observed in solvent controls.

**Table 1:** Larvicidal activity of *Myristica fragrans* essential oil (MFEO) and the synthetic organophosphate temephos against third-instar larvae of *Aedes albopictus* in a modified WHO larval bioassay.

Treatment	Concentration		Mortality (%)				
	ppm	% (w/v)	30 min	60 min	90 min	120 min	24 h
***Myristica fragrans* essential oil (MFEO)**							
MFEO	1000	0.1	100	100	100	100	100
MFEO	500	0.05	100	100	100	100	100
MFEO	100	0.01	100	100	100	100	100
MFEO	50	0.005	90	100	100	100	100
MFEO	10	0.001	80	95	100	100	100
MFEO	1	0.0001	70	80	97	100	100
**Temephos (positive control)**							
Temephos	1000	0.1	100	100	100	100	100
Temephos	500	0.05	100	100	100	100	100
Temephos	100	0.01	100	100	100	100	100
Temephos	50	0.005	100	100	100	100	100
Temephos	10	0.001	90	100	100	100	100
Temephos	1	0.0001	80	90	99	100	100
Solvent control	–	ethanol ≤ 1% v/v	0	0	0	0	0

*Note*: Values are mean percent mortality of three independent replicates. –, not applicable.

*Abbreviation*: ppm, parts per million (w/v).

Because all tested concentrations produced high mortality at 30 min and reached complete mortality by 120 min, probit analysis of the 30-min data yielded low estimated LC_50_ values for both MFEO and temephos. The estimated 30-min LC_50_ was 0.000031% (0.31 ppm; 95% CI: 0.15–0.63 ppm) for MFEO and 0.000014% (0.14 ppm; 95% CI: 0.06–0.34 ppm) for temephos. However, because mortality at the lowest tested concentration was already high, these values should be interpreted as extrapolated indicators of strong larvicidal activity rather than fully bracketed empirical LC_50_ estimates. The overlapping confidence intervals suggest broadly similar larvicidal performance under these laboratory conditions. No delayed recovery or mortality was observed at 24 h.

### 3.3 Adulticidal properties

**Fig. 3:**
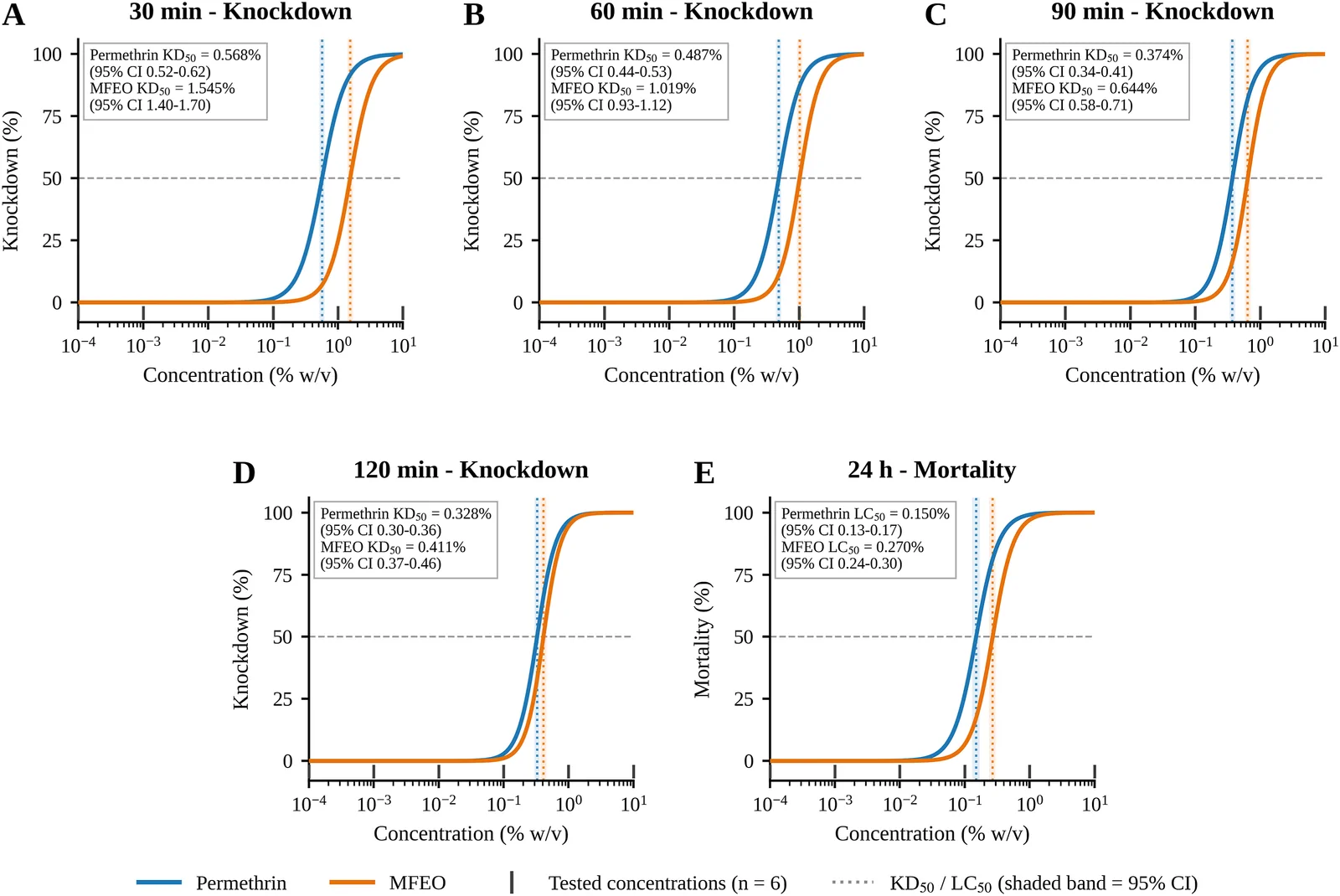
Concentration-response curves for knockdown and mortality of adult *Aedes albopictus* exposed to permethrin and MFEO. Knockdown (KD) was recorded at 30, 60, 90, and 120 min (**A**-**D**), and 24-h mortality (LC) was recorded after a 24-h recovery period with sugar water (**E**). Curves were fitted by non-linear regression using a four-parameter log-logistic model (variable slope) on mean knockdown/mortality percentages from three independent replicates. Median knockdown concentrations (KD_50_) and median lethal concentrations (LC_50_) with their 95% confidence intervals were interpolated from the fitted curves (see Table 2). Goodness-of-fit was assessed by the coefficient of determination (*R*^2^ > 0.90 for all curves) and, where appropriate, by a lack-of-fit F-test (all *P* > 0.05). Symbols represent mean ± SEM; where error bars are not visible, they are smaller than the symbol. The concentration range (10% to 0.0001%) encompassed the full sigmoidal response for all compounds, with the lowest concentration producing < 5% effect.

Adult *Ae. albopictus* responded to both permethrin and MFEO in a clear concentration- and time-dependent manner (Fig. 3, Table 2). For every compound and at every time point, the fitted 4-parameter log-logistic curves displayed the sigmoidal shape, with the response rising sharply across one to two orders of magnitude of concentration. Goodness-of-fit was high in all cases (*R*^2^ > 0.90), and the lack-of-fit F-tests were non-significant (*P* > 0.05), confirming that the log-logistic model adequately described the data. The lowest tested concentration (0.0001%) produced negligible effects (< 5% knockdown or mortality) for both substances, demonstrating that the assay range extended well below the threshold of biological activity and allowed reliable interpolation of the KD_50_ and LC_50_ values.

**Table 2:** Median knockdown (KD_50_) and median lethal (LC_50_) concentrations of permethrin and *Myristica fragrans* essential oil (MFEO) against adult *Aedes albopictus* in WHO tube bioassays.

Time-point	Endpoint	Permethrin (95% CI) (%)	MFEO (95% CI) (%)
30 min	KD_50_	0.568 (0.52–0.62)	1.545 (1.40–1.70)
60 min	KD_50_	0.487 (0.44–0.53)	1.019 (0.93–1.12)
90 min	KD_50_	0.374 (0.34–0.41)	0.644 (0.58–0.71)
120 min	KD_50_	0.328 (0.30–0.36)	0.411 (0.37–0.46)
24 h	LC_50_	0.150 (0.13–0.17)	0.270 (0.24–0.30)

*Abbreviation*: CI, confidence interval.

**Fig. 4:**
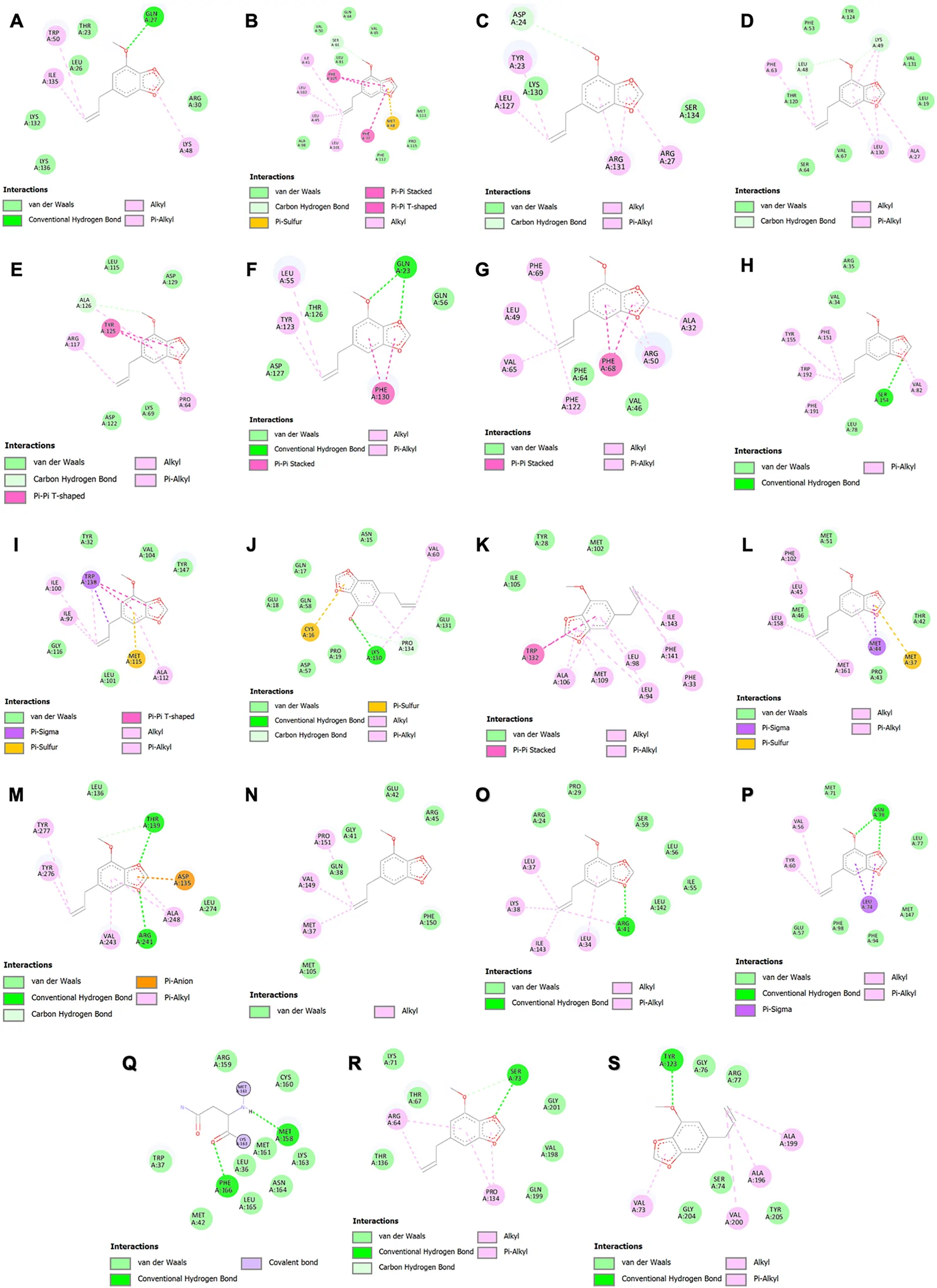
Two-dimensional interaction diagrams of predicted molecular docking interactions between myristicin (PubChem CID 4276) and 19 annotated odorant-binding proteins (OBPs) of *Aedes albopictus*. For each complex, the most thermodynamically favourable binding pose is shown, with myristicin positioned within the receptor binding pocket. Intermolecular interactions (van der Waals, hydrophobic, polar hydrogen) were classified and rendered in BIOVIA Discovery Studio 2019. Panels: (**A**) OBP1, (**B**) OBP3, (**C**) OBP5, (**D**) OBP6, (**E**) OBP10, (**F**) OBP11, (**G**) OBP13, (**H**) OBP24, (**I**) OBP37, (**J**) OBP38, (**K**) OBP39, (**L**) OBP42, (**M**) OBP46, (**N**) OBP55, (**O**) OBP56, (**P**) OBP59, (**Q**) OBP62, (**R**) OBP67, (**S**) OBP68.

Knockdown increased progressively with exposure time, as reflected by the leftward shift of the curves from panel A (30 min) through panel D (120 min) in Fig. 3. For permethrin, the KD_50_ decreased from 0.568% (95% CI: 0.52–0.62%) at 30 min to 0.328% (95% CI: 0.30–0.36%) at 120 min, a 1.7-fold reduction. The decline was even more pronounced for MFEO, whose KD_50_ fell from 1.545% (95% CI: 1.40–1.70%) at 30 min to 0.411% (95% CI: 0.37–0.46%) at 120 min, representing a 3.8-fold reduction over the same interval. Mortality recorded after the 24 h recovery period (Fig. 4E) yielded the lowest median values for both compounds (LC_50_ = 0.150% for permethrin and 0.270% for MFEO), consistent with the cumulative and largely irreversible nature of the lethal endpoint.

Permethrin was the more potent adulticide at every time point, but the potency gap narrowed substantially with prolonged exposure. At 30 min, MFEO required a 2.7-fold higher concentration than permethrin to achieve 50% knockdown (1.545% *vs* 0.568%). By 60 min, the ratio had decreased to 2.1-fold (1.019% *vs* 0.487%); at 90 min the ratio decreased to 1.7-fold (0.644% *vs* 0.374%), and at 120 min the two compounds had converged to a 1.25-fold difference (0.411% *vs* 0.328%). The non-overlapping 95% confidence intervals at 30, 60, and 90 min confirm that these differences are statistically meaningful, whereas the partial overlap at 120 min (permethrin 0.30–0.36 *vs* MFEO 0.37–0.46) indicates that MFEO’s effect approaches that of permethrin with sustained contact. A similar pattern was observed for lethality: the 24 h LC_50_ values differed by less than two-fold (1.8-fold; 0.270% *vs* 0.150%), with their confidence intervals approaching but not overlapping.

Taken together, the curves in Fig. 3 demonstrate that while permethrin acts faster and at lower concentrations, MFEO produces substantial knockdown and lethal effects within the same percent-concentration range and effectively catches up to the synthetic pyrethroid as exposure time increases, supporting its potential as a botanical adulticide against *Ae. albopictus*.

### 3.4 Oviposition deterrent activity

No eggs were deposited on MFEO-treated ovitraps across all tested concentrations (1–0.001%). In contrast, control traps consistently attracted 78–90 eggs. These findings indicate complete oviposition deterrence under the experimental conditions.

### 3.5 The components of *Myristica fragrans* essential oil

MFEO was analyzed through gas chromatography-mass spectrometry (GC-MS), revealing a diverse composition of compounds (Table 3). The most abundant component identified was myristicin, with a retention time of 30.267 min, accounting for 35.35% of the total area. Significant quantities of α-Terpineol (10.74%) and Phenol, 2,4,6-tris(1-phenylethyl)- (11.24%) were also detected at retention times of 20.791 and 44.83 min, respectively. Other major constituents included L-terpinen-4-ol (8.26%) and 2-Phenylpropyl acetate (4.34%). Additionally, compounds present in smaller percentages included p-Cymen-8-ol, trans-Ascaridol glycol, Safrole, p-Menth-2-en-1,4-diol, α-Terpineol acetate, Methyleugenol, Elemicin, Patchoulol, and Dicyclohexano-18-crown-6, with retention times spanning from 20.039 to 44.83 min and area percentages between 0.45% and 2.44%.

**Table 3:** Chemical composition of *Myristica fragrans* essential oil (MFEO) obtained by steam distillation of dried Banda Neira nutmeg kernels and analyzed by gas chromatography-mass spectrometry (GC-MS).

Peak no.	Name/Synonym	Ret. time	Area	Area (%)
1	3-cyclohexen-1-ol,4-methyl-1-(1-methylethyl)-,(R)-/L-terpinen-4-ol	20.039	23778858	8.26
2	Benzenemethanol, α,α4-trimethyl-/p-Cymen-8-ol	20.420	2320626	0.81
3	α-terpineol	20.791	30921412	10.74^a^
4	trans-Ascaridol glycol	23.808	3152356	1.10
5	Safrole	24.186	4900195	1.70
6	p-Menth-2-en-1,4-diol	24.435	3585771	1.25
7	α-Terpinyl acetate	25.914	1285752	0.45
8	Methyleugenol	27.336	1304626	0.45
9	1,3-Benzodioxole, 4-methoxy-6-(2-propenyl)-/Myristicin	30.267	1.02E+08	35.35^a^
10	Benzene, 1,2,3-trimethoxy-5-(2-propenyl)-/Elemicin	30.707	4748400	1.65
11	Patchoulol/Patchouli alcohol	33.343	7025990	2.44
12	Dicyclohexano-18-crown-6	38.835	5722298	1.99
19	Benzeneethanol, β-methyl-, acetate/2-Phenylpropyl acetate	44.215	12504263	4.34
20	Phenol, 2,4,6-tris(1-phenylethyl)-	44.830	32362751	11.24^a^

*Abbreviations*: Ret. time, retention time. NIST, National Institute of Standards and Technology.

a Compounds present at ≥ 10% of total peak area.

### 3.6 Molecular docking of myristicin to odorant-binding proteins

**Fig. 5:**
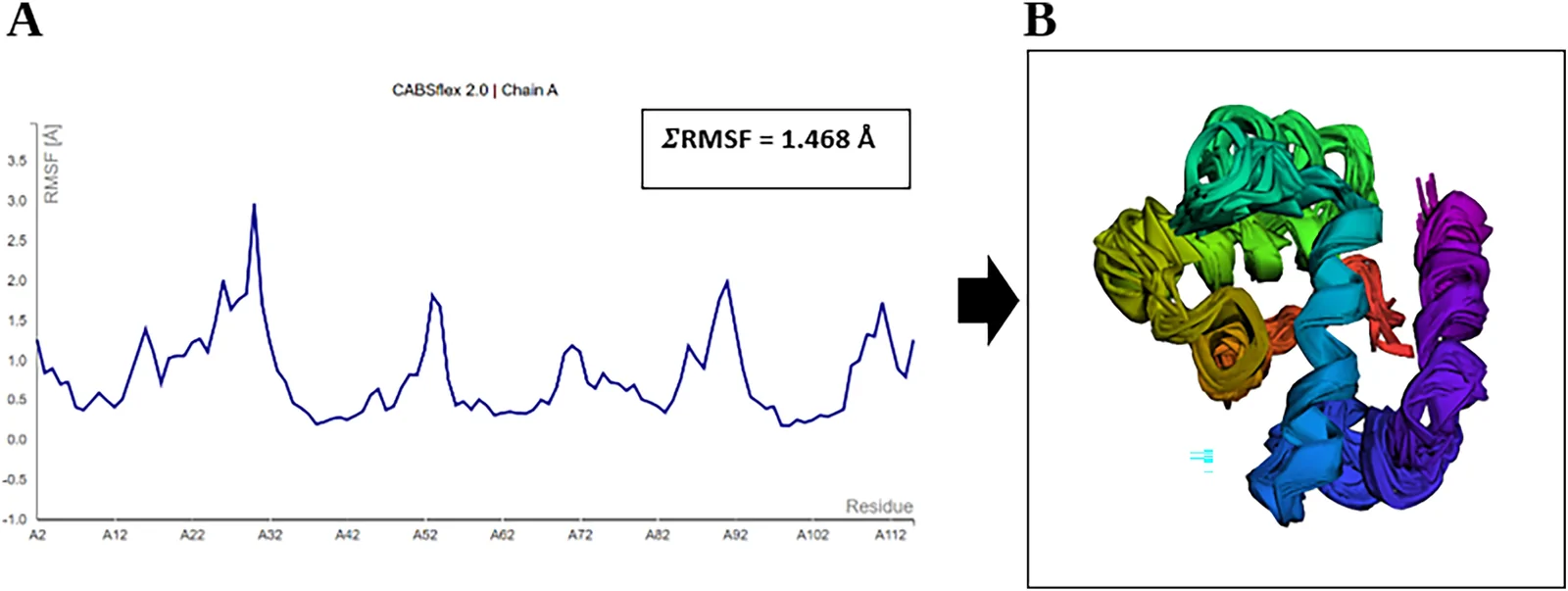
Molecular dynamics (MD) simulation-based validation of the myristicin-OBP3 complex. **A** Per-residue root-mean-square fluctuation (RMSF) profile of Cα atoms across the simulated trajectory; mean RMSF = 1.468 Å. **B** Representative three-dimensional structure of the complex, illustrating overall conformational stability over the simulation.

The results of molecular docking using myristicin compounds against several odorant-binding protein receptors show mixed results. The more negative the binding affinity value of the ligand-receptor complex, the more stable the complex bond in the body (Herdiansyah et al., 2024). The results of molecular docking simulations in Table 4 and Fig. 4 show that the myristicin compound can bind best to the OBP3 receptor (−6.8 kcal/mol). Meanwhile, the lowest binding ability of the myristicin compound is shown in the OBP1 receptor (−4.4 kcal/mol). The chemical bonds possessed by the myristicin-OBP3 complex are van der Waals bonds, hydrogen bonds, and hydrophobic bonds. The myristicin-OBP3 complex that has the strongest binding affinity value was further analyzed using CABS-flex (Fig. 5). The average root mean square deviation (RMSF) value of the complex was 1.468 Å. This value is below the safe threshold for molecular docking validation value of < 2 Å (Aini et al., 2024).

**Table 4:** Predicted molecular docking results for myristicin bound to 19 annotated odorant-binding proteins (OBPs) of *Aedes albopictus*. Molecular docking analysis of myristicin-OBP complexes, including binding affinities and predicted interaction residues. Binding energies (kcal/mol) indicate the strength of ligand-receptor interactions, while interacting amino acid residues are categorized based on van der Waals (vdW), hydrophobic interactions (HI), and polar hydrogen interactions (PHI).

Complex ligand-receptor	Complex binding affinity (kcal/mol)	Type of interaction	Amino acids involved
Myristicin-OBP1	−4.4	vdW	Lys132(A), Lys136(A), Leu26(A), Thr23(A), Arg30(A)
		HI	Lys48(A), Ile135(A), Trp50(A)
		PHI	Gln27(A)
Myristicin-OBP3	−6.8	vdW	Gln64(A), Val65(A), Leu81(A), Val50(A), Ala98(A), Met113(A), Pro115(A), Phe112(A)
		HI	Leu101(A), Leu45(A), Leu102(A), Ile41(A)
		PHI	Ser61(A)
Myristicin-OBP5	−4.7	vdW	Ser134(A), Lys130(A)
		HI	Tyr23(A), Arg27(A), Leu127(A), Arg131(A)
		PHI	Asp24(A)
Myristicin-OBP6	−5.7	vdW	Val67(A), Ser64(A), Thr120(A), Phe53(A), Tyr124(A), Val131(A), Leu19(A)
		HI	Ala27(A), Phe63(A), Leu130(A)
		PHI	Leu48(A), Lys49(A)
Myristicin-OBP10	−4.6	vdW	Leu115(A), Asp129(A), Lys69(A), Asp122(A)
		HI	Pro64(A), Arg117(A)
		PHI	Ala126(A)
Myristicin-OBP11	−6.2	vdW	Gln56(A), Thr126(A), Asp127(A)
		HI	Leu55(A), Tyr123(A)
		PHI	Gln23(A)
Myristicin-OBP13	−6.1	vdW	Val46(A), Phe64(A)
		HI	Ala32(A), Arg50(A), Phe122(A), Val65(A), Leu49(A), Phe69(A)
Myristicin-OBP46	−5.4	vdW	Leu136(A), Leu274(A)
		HI	Ala248(A), Val243(A), Tyr276(A), Tyr277(A)
		PHI	Thr139(A), Arg241(A)
Myristicin-OBP24	−6.0	vdW	Leu78(A), Val34(A), Arg35(A)
		HI	Val82(A), Phe191(A), Trp192(A), Phe151(A), Tyr155(A)
		PHI	Ser154(A)
Myristicin-OBP37	−6.4	vdW	Tyr32(A), Val104(A), Tyr147(A), Gly116(A), Leu101(A)
		HI	Ile97(A), Ile100(A), Ala112(A)
Myristicin-OBP38	−5.3	vdW	Glu131(A), Asn15(A), Pro19(A), Asp57(A), Gln58(A), Gln17(A), Glu18(A)
		HI	Val60(A)
		PHI	Lys130(A), Pro134(A)
Myristicin-OBP39	−6.5	vdW	Met102(A), Tyr28(A), Ile105(A)
		HI	Ile143(A), Phe141(A), Phe33(A), Leu94(A), Leu98(A), Met109(A), Ala106(A)
Myristicin-OBP42	−4.6	vdW	Met51(A), Met46(A), Thr42(A), Pro43(A)
		HI	Met161(A), Leu158(A), Leu45(A), Phe102(A)
Myristicin-OBP55	−4.5	vdW	Arg45(A), Phe150(A), Glu42(A), Gly41(A), Gln48(A), Met105(A)
		HI	Pro151(A), Val149(A), Met37(A)
Myristicin-OBP56	−5.1	vdW	Leu142(A), Ile55(A), Leu56(A), Ser59(A), Pro29(A), Arg24(A)
		HI	Leu37(A), Lys38(A), Ile143(A), Leu34(A)
		PHI	Arg41(A)
Myristicin-OBP59	−5.4	vdW	Met71(A), Leu77(A), Met147(A), Phe94(A), Phe98(A), Glu57(A)
		HI	Tyr60(A), Val56(A)
		PHI	Asn78(A)
Myristicin-OBP62	−5.5	vdW	Arg159(A), Cys160(A), Met161(A), Leu36(A), Lys163(A), Asn164(A), Leu165(A), Met42(A), Trp37(A)
		PHI	Phe166(A), Met158(A)
Myristicin-OBP67	−5.2	vdW	Lys71(A), Thr67(A), Thr136(A), Gly201(A), Val198(A), Gln199(A)
		HI	Arg64(A), Pro134(A)
		PHI	Ser73(A)
Myristicin-OBP68	−5.2	vdW	Gly76(A), Arg77(A), Ser74(A), Gly204(A), Tyr205(A)
		HI	Ala199(A), Ala196(A), Val200(A), Val73(A)
		PHI	Tyr123(A)

*Abbreviations*: vdW, van der Waals interaction; HI, hydrophobic interaction; PHI, polar hydrogen interaction.

### 3.7 Comparative docking against neurophysiological targets and DEET

**Fig. 6:**
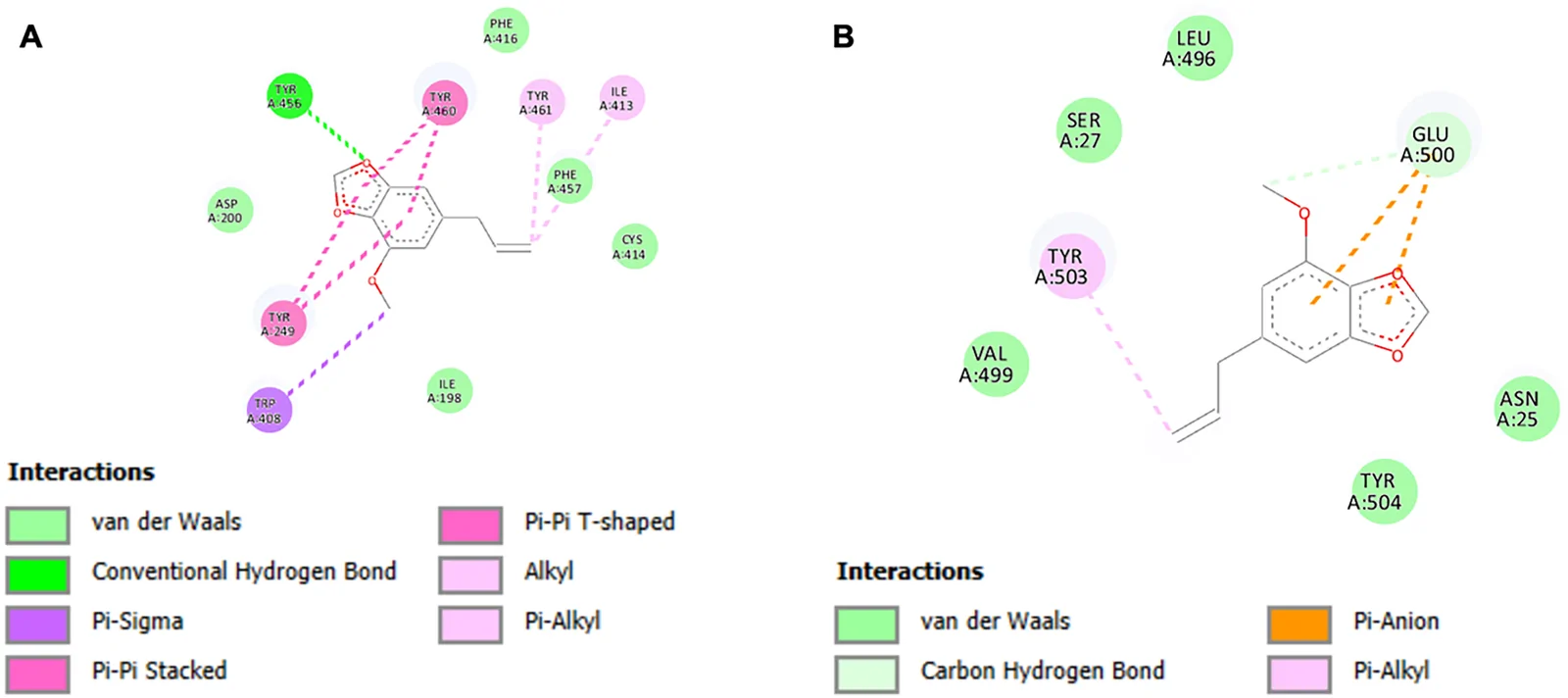
Molecular docking interactions between myristicin and two cholinesterase isoforms of *Aedes albopictus*. **A** Canonical acetylcholinesterase (AChE) (binding affinity of −6.3 kcal/mol). **B** AChE-like/butyrylcholinesterase-like protein (binding affinity of −4.7 kcal/mol). Key interacting residues, including Tyr456(A) and Tyr461(A) in AChE (*via* HI and vdW), are annotated, with full interaction profiles reported in Supplementary Table S4.

**Fig. 7:**
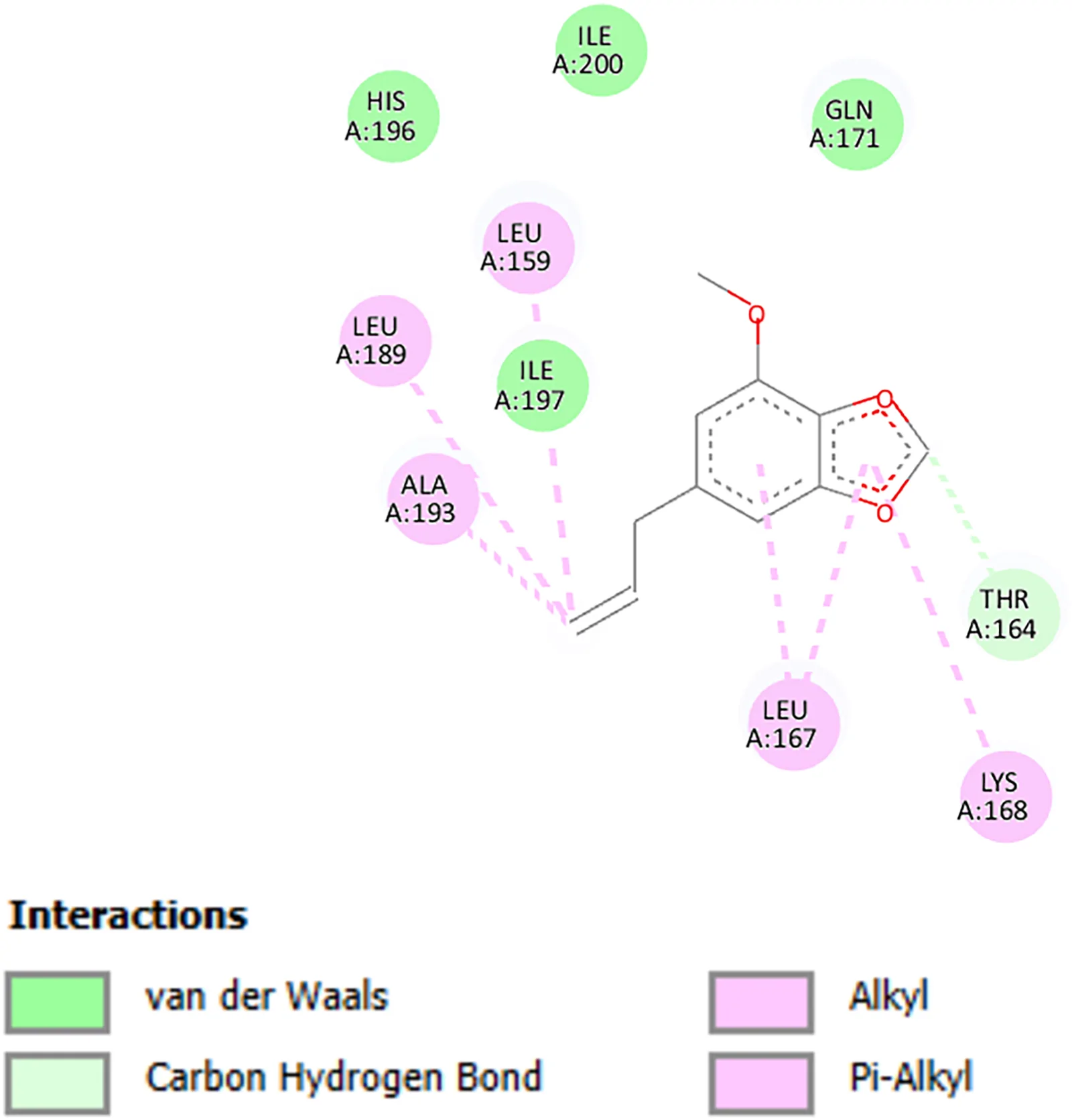
Predicted docking complex between myristicin and the voltage-gated sodium channel (VGSC/para) of *Aedes albopictus* (binding affinity of −5.3 kcal/mol). VGSCs are the primary molecular targets of pyrethroid and DDT-type insecticides, where ligand binding typically prolongs channel opening and induces repetitive neuronal firing. No polar hydrogen bonds were observed in the most stable pose.

**Fig. 8:**
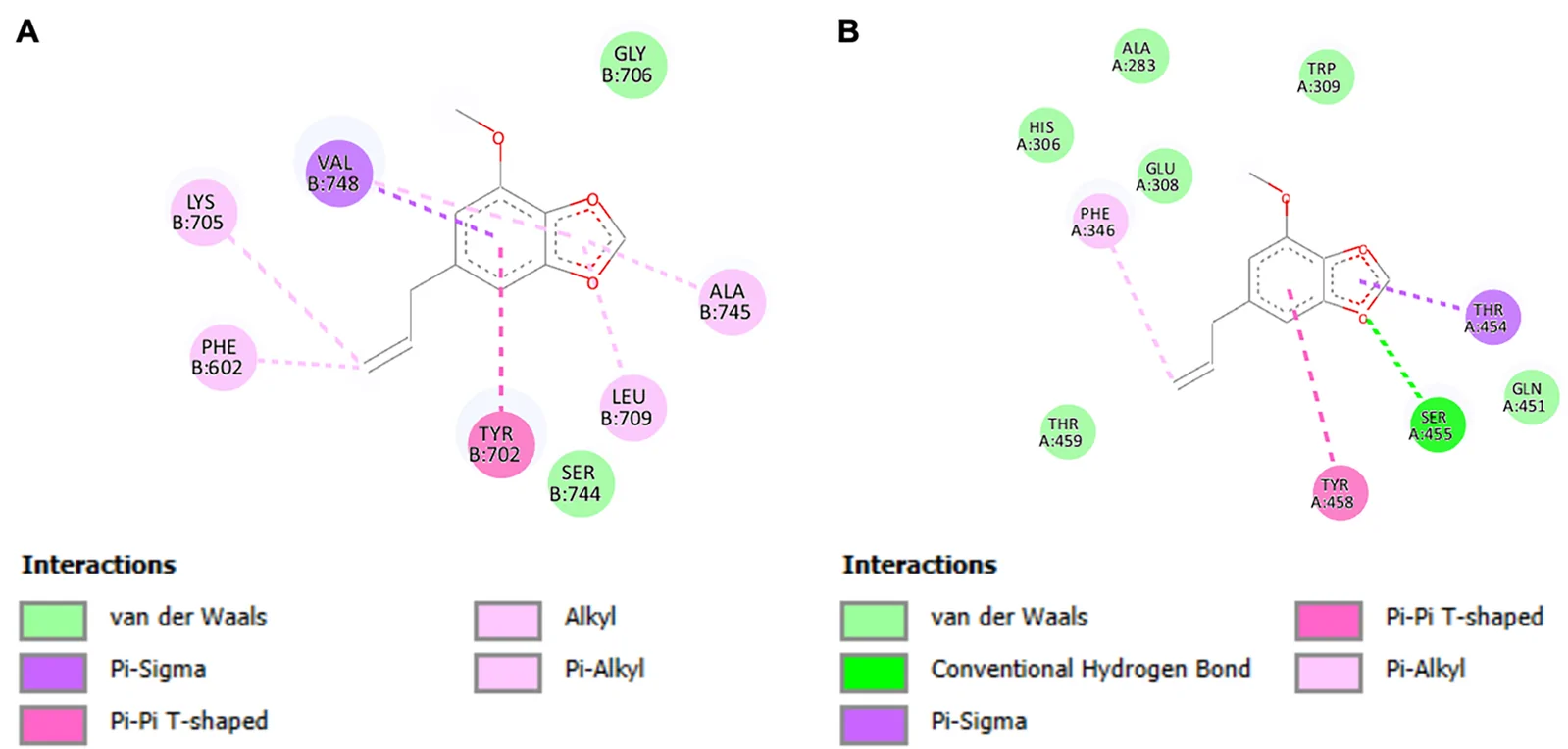
Predicted binding conformations of myristicin with two gamma-aminobutyric acid (GABA) receptor subunits of *Aedes albopictus*. **A** GABA-B-R2 (binding affinity of −6.0 kcal/mol). **B** GABA-A α-6 subunit (binding affinity of −5.6 kcal/mol). GABA receptors mediate inhibitory neurotransmission in arthropods; their disruption leads to hyperexcitation, convulsions, and mortality. Myristicins affinity for GABA-B-R2, stabilized by hydrophobic residues (Lys705(A), Phe602(A)) and van der Waals contacts (Gly706(A), Ser744(B)), suggests potential allosteric modulation. In contrast, interaction with the GABA-A α-6 subunit involves a broader polar and van der Waals network (e.g. Glu308(B), His306(B), Ser455(B)), indicating possible competitive or non-competitive antagonism.

**Fig. 9:**
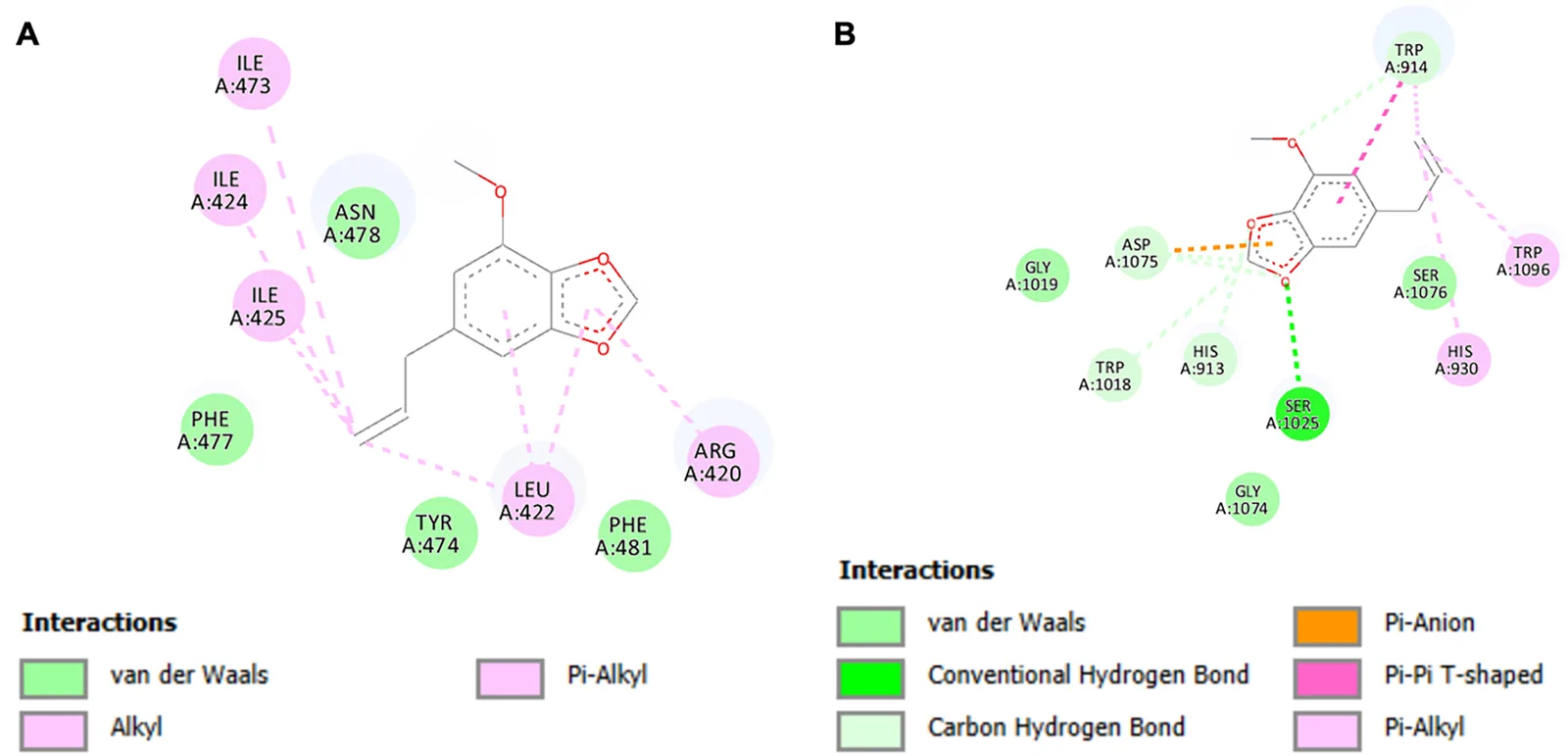
Molecular docking profiles of myristicin with two octopamine receptor (OR) isoforms in *Aedes albopictus*. **A** Octβ2R (binding affinity of −4.9 kcal/mol); myristicin is held by hydrophobic contacts (Arg420(A), Leu422(A), Ile424(A), Ile425(A), Ile473(A)) and van der Waals contacts (Tyr474(A), Phe477(A), Asn478(A), Phe481(A)), with no hydrogen bonding. **B** Octβ2R-like (binding affinity of −5.6 kcal/mol); binding is driven by hydrophobic contacts (His930(A), Trp1096(A)) and van der Waals contacts (Gly1019(A), Gly1074(A), Ser1076(A)), together with a conventional hydrogen bond to Ser1025(A).

Beyond the odorant-binding proteins, myristicin was docked against four classes of neurophysiological target proteins of *Ae. albopictus* (Supplementary Tables S2 and S3). Myristicin bound both cholinesterase isoforms, showing higher affinity for canonical acetylcholinesterase (AChE; −6.3 kcal/mol) than for the AChE-like/butyrylcholinesterase-like protein (−4.7 kcal/mol) (Fig. 6). A moderate affinity was predicted for the voltage-gated sodium channel (VGSC/para; −5.3 kcal/mol), mediated predominantly by hydrophobic interactions (Leu189(A), Ala193(A)) and van der Waals contacts (His196(A), Ile197(A)), with no polar hydrogen bonds in the most stable pose (Fig. 7). Among the GABA receptors, myristicin bound the GABA-B-R2 subunit (−6.0 kcal/mol) and the GABA-A α-6 subunit (−5.6 kcal/mol) (Fig. 8), while among the octopamine receptors it bound the Octβ2R-like isoform (−5.6 kcal/mol) more strongly than Octβ2R (−4.9 kcal/mol) (Fig. 9). Full residue-level interaction profiles for all neurophysiological complexes are provided in Supplementary Table S4.

For comparison, the reference synthetic repellent N,N-diethyl-3-methylbenzamide (DEET) was docked against the same panel of molecular targets (Table 5). The three-dimensional docking of DEET against the 19 OBPs and the neurophysiological targets is shown in Supplementary Fig. S2, and the corresponding residue-level binding profile across the OBP panel is reported in Supplementary Table S5. The side-by-side summary in Table 5 allows the DEET binding affinities and interacting residues to be compared directly with the myristicin profiles across the OBP, VGSC, GABA-receptor, and octopamine-receptor target classes.

**Table 5:** Comparative summary of N,N-diethyl-3-methylbenzamide (DEET) docking results across the four principal classes of *Aedes albopictus* molecular targets investigated in this study: odorant-binding proteins (OBPs), voltage-gated sodium channels (VGSCs), GABA receptors (GABARs), and octopamine receptors (ORs). Binding affinities (kcal/mol) and interacting residues for the most favourable pose per complex are summarised side-by-side to facilitate cross-target comparison of DEET, and to allow side-by-side comparison with the corresponding myristicin profiles reported in Table 4.

Complex ligand-receptor	Complex binding affinity (kcal/mol)	Type of interaction	Amino acids involved
DEET-AChE	−6.1	vdW	Gly412(A), Glu415(A), Ile413(A), Cys414(A), Tyr461(A), Ile198(A), Tyr456(A), Val202(A)
		HI	Tyr460(A), Trp408(A), Phe416(A)
		PHI	Tyr249(A)
DEET-AChE-like	−5.1	vdW	Leu512(A), Ala15(A)
		HI	Ile18(A), Pro14(A), Trp513(A), Pro509(A)
		PHI	–
DEET-GABA-B-R2	−6.0	vdW	Leu701(B), Ser744(B)
		HI	Tyr702(B), Lys705(B), Leu598(B)
		PHI	Ser599(B)
DEET-GABA-A alpha-6	−6.5	vdW	Met370(A), Tyr449(A), Thr450(A), Thr454(A), Asn446(A)
		HI	Lys371(A)
		PHI	–
DEET-VGSC/para	−5.7	vdW	Arg106(A), Asn107(A), Val151(A), Asp152(A), Thr73(A), Gln163(A)
		HI	Met70(A), Leu105(A), Leu78(A)
		PHI	–
DEET-Octβ2R	−5.1	vdW	Asn478(A), Tyr474(A), Ile473(A), Ile425(A)
		HI	Phe477(A)
		PHI	Gly423(A), Ile424(A)

*Abbreviations*: vdW, van der Waals interaction; HI, hydrophobic interaction; PHI, polar hydrogen interaction.

## 4 Discussion

### 4.1 Ovicidal of *Myristica fragrans* essential oil on *Aedes albopictus*

Mosquito eggs are widely recognized as one of the most resilient and difficult life stages to eliminate, yet they remain relatively understudied as targets for chemical or botanical control measures (Farnesi et al., 2015; Campbell et al., 2016). Their remarkable resistance is largely attributed to a specialized multilayered eggshell structure. The chorion, composed of the exochorion and endochorion, is formed during choriogenesis by follicular epithelial cells and is already present at oviposition. During early embryogenesis, an additional protective layer, the serosal cuticle, is synthesized by the extraembryonic serosa and deposited beneath the endochorion (Rezende et al., 2008; Farnesi et al., 2017). Together, these layers form a highly specialized barrier system that confers mechanical stability, regulates gas exchange, and minimizes water loss. The exochorion exhibits species-specific microstructures such as meshworks, tubercles, aeropyles, and papillae, which contribute to controlled permeability and environmental adaptation (Campbell et al., 2016). The serosal cuticle plays a particularly important role in limiting trans-chorionic water flux, allowing eggs to withstand prolonged dry periods and remain viable for months, sometimes exceeding one year (Farnesi et al., 2017; Mundim-Pombo et al., 2021).

From a control perspective, the chitin-protein matrix of the eggshell presents both a challenge and an opportunity. Chitin, a critical structural component of the serosal cuticle, varies among species and substantially contributes to eggshell rigidity and environmental tolerance (Farnesi et al., 2015). While this multilayered barrier provides strong resistance against environmental insults and many conventional insecticides, disruption of the exochorion or interference with serosal cuticle integrity can compromise structural stability, increase permeability, and lead to embryonic mortality. Compounds capable of destabilizing lipid-protein interactions or altering chitin organization may exert ovicidal effects by enhancing porosity, promoting desiccation, or impairing embryonic gas exchange. The ovicidal activity of MFEO is most plausibly attributed to its oxygenated monoterpenes, particularly α-terpineol and terpinen-4-ol, which are known to disrupt lipid-protein matrices and enhance membrane permeability. These amphiphilic compounds likely penetrate the exochorionic layer, destabilize chitin-associated structural proteins, and increase porosity, thereby impairing embryonic respiration and promoting desiccation-induced mortality (Fig. 1). This mechanism, also observed in other ovicides, involves interactions with chitin solubilizing or weakening its lipid and protein matrices, thereby increasing the porosity of the eggshell (Li et al., 2021; Moungthipmalai et al., 2023; Passara et al., 2024). Some investigations have demonstrated that the terpenes α-terpineol and terpinen-4-ol possess significant ovicidal activity against a range of arthropod pests. Studies on the human head louse (*Pediculus humanus capitis*) have shown that terpinen-4-ol causes 94% and 69% inhibition of egg hatch at concentrations of 0.25 and 0.125 mg/cm^2^, respectively, while α-terpineol achieves 88% and 76% inhibition at 0.5 and 0.25 mg/cm^2^ (Yang et al., 2009). Against the eggs of the scabies mite (*Sarcoptes scabiei*), terpinen-4-ol exhibits an EC_50_ (median effective concentration for 50% egg mortality) of 5.1%, with microscopic examination suggesting that these terpenes may act by penetrating through the aeropyles, specialized microstructures, on the eggshell surface (Li et al., 2021). This proposed mechanism of action aligns directly with the structural composition of the insect eggshell, where such microstructures facilitate gas exchange but may also serve as entry points for ovicidal compounds. The insecticidal properties of these monoterpenoids have been well documented, with α-terpineol and its isomer terpinen-4-ol recognized as the most common terpineols found in nature (Khaleel et al., 2018). Altogether, these interactions weaken the eggshell defences, rendering the egg more vulnerable to environmental stressors and leading to embryonic mortality (Kingsolver et al., 2011; Campbell et al., 2016).

### 4.2 Larvicidal efficacy of *Myristica fragrans* essential oil against *Aedes albopictus*

The comparative evaluation of MFEO and temephos against *Ae. albopictus* larvae revealed clear concentration- and time-dependent differences in larvicidal performance. At higher concentrations, both MFEO and temephos induced complete larval mortality within 30 min and sustained 100% mortality throughout the 24-h observation period. This indicates that, at sufficiently high doses, MFEO exhibits larvicidal efficacy comparable to that of the conventional synthetic larvicide temephos.

The primary active compound in MFEO, myristicin (35.35%), is a well-documented insecticide and synergist. This aligns with a study evaluating naturally occurring compounds against *Culex pipiens pallens* larvae, which found that myristicin exhibited potent larvicidal activity with an LC_50_ value of 0.56 μg/ml. The methylenedioxy moiety myristicin might involve lowering the water surface tension, thereby interfering with larval respiration (Bae et al., 2017). Further strengthening the case for a multi-modal action, studies have shown that phenylpropanoids like myristicin can be detoxified by insect cytochrome P450 enzymes, a process that can lead to the production of even more toxic intermediates. This metabolic interference explains the “slower onset of mortality” we observed at lower concentrations, as the toxic effect relies on the larvae’s own metabolic processes. While α-terpineol (10.74%) and L-terpinen-4-ol (8.26%) of *Origanum vulgare* essential oil showed significant toxicity against several mosquito vectors, including *Anopheles stephensi* (LC_50_ = 43.27 μg/ml) and *Culex quinquefasciatus* (LC_50_ = 52.19 μg/ml) (Govindarajan et al., 2016). The terpenes (α-terpineol and terpinen-4-ol) are known for their rapid physical and neurotoxic effects, such as disrupting cell membranes and inhibiting acetylcholinesterase, leading to quicker paralysis and death (Govindarajan et al., 2016). The combination of these slow-acting phenylpropanoids and fast-acting terpenes in a single essential oil creates a powerful synergistic effect. This synergy has been observed in other studies where mixtures of terpenes and phenylpropanoids from essential oils induced significant biochemical changes, oxidative stress, and morphological damage in insects, resulting in higher overall toxicity than any single compound alone (Hummelbrunner and Isman, 2001; Scalerandi et al., 2018).

The slower onset of mortality observed for MFEO at lower concentrations may reflect differences in modes of action between botanical and synthetic larvicides. Temephos exerts rapid neurotoxic effects through acetylcholinesterase inhibition, leading to fast larval paralysis and death (Martínez-Mercado et al., 2022). In contrast, essential oils are complex mixtures of multiple bioactive compounds that may act through diverse mechanisms, including interference with metabolic pathways and impairment of sensory or respiratory functions (Silva et al., 2024). Such multi-target interactions may result in a more gradual increase in mortality while still producing complete lethality with sufficient exposure duration. Despite a slightly slower action at the lowest concentrations, MFEO achieved full larval mortality across all tested doses, supporting its potential as an effective botanical larvicide. Further studies evaluating residual activity, formulation stability, and field efficacy would be valuable to better define its operational potential.

### 4.3 Adulticidal activity of *Myristica fragrans* essential oil against *Aedes albopictus*

Our study demonstrates that MFEO possesses rapid, time-dependent knockdown and lethal activities against adult *Ae. albopictus*. The rapid knockdown observed with MFEO can be attributed to its complex composition of neuroactive monoterpenoids and phenylpropenes. *Myristica fragrans* has previously shown insecticidal potential against several arthropod species, including *Blattella germanica*, *Anthrenus verbasci*, *Musca domestica*, and *Chrysomya albiceps* (Jung et al., 2007; Cossetin et al., 2021). Myristicin inhibits acetylcholinesterase (AChE), leading to synaptic accumulation of acetylcholine and persistent neuronal firing (Seneme et al., 2021). Consistent with this, the higher predicted affinity of myristicin for canonical AChE (−6.3 kcal/mol) than for the AChE-like/butyrylcholinesterase-like isoform (−4.7 kcal/mol) suggests competitive inhibition of acetylcholine hydrolysis at the synaptic cleft, a mechanism analogous to that of organophosphate and carbamate insecticides. It also suppresses cytochrome P450 monooxygenases, which are critical for detoxifying both endogenous and xenobiotic compounds, and this inhibition may potentiate the effect of co-occurring terpenoids (Mao et al., 2008). α-terpineol, another prominent component, potentiates GABA-gated chloride channels, causing hyperpolarisation and paralysis (Khaleel et al., 2018; Spinozzi et al., 2021). Additional constituents such as L-terpinen-4-ol, α-terpineol acetate, methyleugenol, and safrole contribute moderate adulticidal and repellent activities through combined AChE inhibition and induction of oxidative stress. While patchoulol and p-menth-2-en-1,4-diol are primarily known as repellents, their modulation of sensory and neural pathways may act additively to weaken the insect before lethal thresholds are reached (Seneme et al., 2021; Spinozzi et al., 2021). This multi-target neurodisruptive action contrasts with the single voltage-gated sodium channel target of permethrin, and the time-dependent decline in KD_50_ (from 1.545% at 30 min to 0.411% at 120 min) reflects cumulative neurological impairment that eventually becomes irreversible, as shown by the 24-h LC_50_ of 0.270%. Consistent with this, the only moderate predicted affinity of myristicin for the VGSC (−5.3 kcal/mol) points to an auxiliary modulatory effect rather than direct pore blockade; any contribution to knockdown is therefore more plausibly explained by combined action with other neuroactive constituents of MFEO, such as α-terpineol and terpinen-4-ol, than by sodium-channel binding alone.

The progression of median knockdown concentrations over time is a hallmark of essential oils that require penetration and metabolic activation within the insect. For MFEO, the KD_50_ decreased almost four-fold between 30 and 120 min, indicating that its constituents progressively engage multiple targets. In comparison with other plant essential oils tested against *Ae. albopictus* under similar WHO tube conditions, MFEO ranks among the more potent formulations. Recent work by Yongsue and Tainchum (2025) established discriminating concentrations (DCs; producing 98–100% knockdown and 99–100% mortality) for *Litsea cubeba*, *Syzygium aromaticum* (clove), and *Cinnamomum porrectum* essential oils at 9.68%, 24.33%, and 15.55% v/v, respectively. These DCs are ∼36- to 90-fold higher than the 24-h LC_50_ of MFEO (0.270%), highlighting the comparatively high intrinsic lethality of the oil. In fumigation bioassays, *Zingiber cassumunar* essential oil required a 24-h LC_50_ of 5.44% against the same vector (Li et al., 2021), approximately 20-fold greater than the contact LC_50_ reported here. When applied at 5–10% singly, *Cymbopogon citratus* and *Eucalyptus globulus* oils achieved only 32–58% knockdown at 1 h against *Ae. albopictus*; synergistic 1:1 mixtures at 10 + 10% were necessary to reach 100% knockdown (Soonwera and Sittichok, 2020). By contrast, MFEO induced 50% knockdown at just 1.019% within 1 h without any synergist. The 1-h KD_50_ of MFEO (1.019%) is higher than reported topical-application KD_50_ values for clove bud-, clove leaf-, and cinnamon leaf oils against *Ae. aegypti* (0.20–0.21%; Norris et al., 2018), but this difference is expected given that contact exposure in WHO tubes typically yields higher nominal KD_50_ values than direct topical application. The comparison shows that MFEO falls within the effective range of known neuroactive essential oils and is notably more potent than several species that have already reached field-trial stage.

### 4.4 Effects of *Myristica fragrans* essential oil on the oviposition behavior of *Aedes albopictus*

Mosquitoes rely heavily on their olfactory systems to locate suitable oviposition sites. This system comprises odorant-binding proteins (OBPs), odorant receptors (ORs), and olfactory sensory neurons (OSNs), all of which are critical for detecting chemical cues. Odor molecules released from potential oviposition sites or hosts bind to ORs on the dendritic membrane of OSNs, with OBPs facilitating this binding. This interaction triggers neuronal activation, converting external chemical stimuli into electrical signals (Hallem and Carlson, 2006). OBPs play a pivotal role in the sensitive detection of oviposition attractants. Modulating or interfering with OBP function can significantly disrupt a mosquito’s ability to locate and evaluate suitable egg-laying sites (Yan et al., 2022). The potent oviposition-deterrent effects of MFEO observed in this study, across a broad range of concentrations, underscore its efficacy as a repellent against mosquito egg-laying. This deterrence likely stems from the interference of MFEO with the chemosensory signals that mosquitoes rely on to identify suitable oviposition sites. Notably, some substances have shown that specific oviposition cues, such as indole, can interact with olfactory receptors to inhibit host-seeking behavior, thereby altering mosquitoes’ normal behavioral patterns (Dekel et al., 2022). By disrupting the olfactory pathways responsible for site selection, MFEO-treated surfaces become unattractive for egg deposition. Consequently, female mosquitoes in this study demonstrated a clear preference for untreated control cups over those treated with MFEO, highlighting the effectiveness of essential oils in modifying mosquito oviposition behavior. Supporting this, molecular docking results detailed below further elucidate the interaction mechanisms between MFEO components and mosquito olfactory proteins.

Treated filter paper tests consistently showed an absence of egg deposition, in contrast to control traps, which attracted 78–90 eggs. This outcome aligns with findings from other plant-based essential oils, such as *Lantana camara*, *Schinus terebinthifolia*, *Callistemon viminalis*, and *Helichrysum odoratissimum*. These essential oils have demonstrated oviposition deterrence against mosquito species such as *Ae*. *aegypti*, *Anopheles gambiae*, and *Culex quinquefasciatus*, with varying levels of effectiveness (Abbas et al., 2024). Notably, compounds like limonene, β-pinene, and 1,8-cineole have been identified as potent oviposition deterrents, effectively disrupting egg-laying behavior in *Cx*. *quinquefasciatus* (Andrade-Ochoa et al., 2018) and in multiple mosquito species, including *Ae*. *aegypti*, *Anopheles dirus*, and *Cx*. *quinquefasciatus* (Phasomkusolsil and Soonwera, 2012).

The absence of egg deposition also suggests potential implications for integrated vector management. Unlike larvicides that act post-oviposition, oviposition deterrents interrupt the mosquito life cycle at an earlier stage, limiting population establishment in treated habitats. For invasive and peri-urban vectors such as *Ae. albopictus*, which exploit artificial containers, oviposition repellents could be strategically applied to high-risk breeding sites. Moreover, behavioral disruption strategies may exert lower selective pressure for resistance compared to neurotoxic insecticides.

### 4.5 Chemical profile of *Myristica fragrans* essential oil

The chemical composition of MFEO, as revealed by GC-MS analysis, underscores its multifaceted bioactivity against mosquitoes. Instead of many MFEO analyses, we herewith presented the components of MFEO from Banda Neira, where the plant is natively growing. The GC-MS analysis of MFEO obtained from Banda Neira reveals a chemically diverse profile dominated by oxygenated monoterpenes, oxygenated non-terpenes (notably phenylpropanoids), and a smaller proportion of oxygenated sesquiterpenes. Oxygenated monoterpenes such as terpinen-4-ol, α-terpineol, p-cymen-8-ol, p-menth-2-en-1,4-diol, and trans-ascaridol glycol constitute a substantial fraction of the oil. These compounds are widely reported to possess insecticidal and larvicidal properties, primarily through disruption of cellular membranes, interference with respiratory metabolism, and impairment of nervous system function. Their relatively small molecular size and moderate polarity may facilitate rapid penetration through the larval cuticle, contributing to the high mortality observed at short exposure times, particularly at higher concentrations (Andrade-Ochoa et al., 2018). In addition to monoterpenes, MFEO contains several oxygenated non-terpene compounds, including safrole, methyleugenol, myristicin, and elemicin, which belong to the phenylpropanoid class (Abourashed and El-Alfy, 2016). Phenylpropanoids are known for their strong bioactivity against insects, often acting as neurotoxic agents, feeding deterrents, or behavior-modifying compounds. The presence of these compounds may contribute to both acute toxicity and cumulative larvicidal effects observed at lower concentrations, potentially explaining the progressive increase in mortality over time. Importantly, phenylpropanoids have also been implicated in disruption of olfactory and sensory processes in insects, which aligns with the *in silico* docking analysis targeting odorant-binding proteins presented in this study. Patchoulol, an oxygenated sesquiterpene identified in the oil, may further enhance larvicidal efficacy through synergistic interactions with phenylpropanoids (Feng et al., 2019). Sesquiterpenes are often associated with prolonged or residual bioactivity due to their higher molecular weight and lower volatility, which could contribute to sustained larval mortality during extended exposure periods (Portilla-Pulido et al., 2020). Overall, the chemical composition of MFEO reflects a complex mixture of bioactive compounds with complementary and potentially synergistic modes of action.

### 4.6 Molecular docking of myristicin to odorant-binding proteins

The molecular docking experiments show that myristicin exerts different binding effects on odorant-binding protein receptors. The myristicin compound tends to bind more easily to the OBP3 receptor of mosquitoes than others with a binding energy value of −6.8 kcal/mol. Biochemically, molecular docking technique represents the interaction between the ligand and the receptor at the physical and chemical level of the native condition when inside the body. The main interactions shared by the whole complex are van der Waals bonds, hydrogen bonds, and hydrophobic bonds. The specificity of hydrogen bonds allows the ligand to occupy the right amino acid as the main target component for improved drug efficacy (Baker and Hubbard, 1984). Meanwhile, each complex that has a binding free energy value in the molecular docking simulation represents the pharmacological properties of the drug substance when in the body. When the scoring process is performed, each component of binding energy is considered, especially the van der Waals bond (Songtawee et al., 2013). This is done to predict the specification and strength of the drug substance in forming a receptor-ligand complex (Gayathiri et al., 2023). The binding affinity value is also supported by the results of the molecular dynamics value of the myristicin-OBP3 complex with a value of 1.468 Å. This value is below the agreed threshold of molecular dynamics provisions with a value of < 2 Å (Songtawee et al., 2013). The greater the deviation value of molecular dynamics, the greater the error value in predicting ligand-receptor interactions (Yang et al., 2022).

The combined oviposition-deterrent, ovicidal, larvicidal, and adulticidal activities of MFEO, supported by molecular docking evidence for interaction with OBPs, position this botanical extract as a multi-stage intervention candidate for *Ae. albopictus* control. However, we recognise that translating these laboratory findings into operational field use is currently constrained by the inherent volatility of essential oils and the absence of formulation and stability data. Future work must therefore prioritise formulation development, such as microencapsulation, slow-release matrices, or polymer-based surface coatings, to enhance residual activity on container walls in high-risk oviposition sites (e.g. discarded tyres, peri-domestic containers, water storage vessels), where *Ae. albopictus* deposits drought-resistant eggs capable of long-distance dispersal. Semi-field and field validation studies are essential to evaluate efficacy under fluctuating environmental conditions (rainfall, temperature, UV exposure) and to assess oviposition deterrence under natural population pressure, as well as compatibility with existing vector management tools such as source reduction and larval control. Additionally, the observed interaction between myristicin and OBP3 suggests that MFEO constituents may function not only as toxicants but also as behavioural disruptors, opening opportunities for “push-pull” strategies in which MFEO-treated surfaces act as the “push” component to deter egg-laying in critical areas, while attractant-baited traps serve as the “pull” to concentrate oviposition in controlled kill zones. Systematic ecotoxicological assessment is also required before field deployment, as the assumption of lower non-target toxicity, while plausible for a plant-derived product, has not yet been tested. These sequential development steps will determine whether MFEO can be integrated as a complementary tool within integrated vector management programmes, reducing reliance on conventional neurotoxic insecticides and potentially mitigating resistance-selective pressure.

Although the predicted binding energies for the octopamine receptor isoforms (−4.9 and −5.6 kcal/mol) are moderate relative to that for OBP3 (−6.8 kcal/mol), such interactions may contribute to sublethal behavioural effects, including reduced host-seeking and oviposition deterrence, and to metabolic stress, particularly in combination with other MFEO constituents. The multi-target activity of MFEO, potentially affecting neural pathways, detoxification enzymes, eggshell integrity, and chemosensory proteins, may collectively contribute to the observed adulticidal, ovicidal, larvicidal, and oviposition-deterrent effects. This integrated action suggests a reduced likelihood of rapid resistance development compared with single-target synthetic insecticides, although systematic resistance monitoring and long-term ecological impact assessments remain necessary. From a sustainability perspective, the use of locally sourced MFEO, particularly from regions where *M. fragrans* is cultivated, may offer economic and environmental advantages; however, large-scale production, standardisation of chemical profiles, and comprehensive safety evaluation for non-target organisms and humans must be addressed before commercialisation.

The multi-stage activity observed in this study suggests that MFEO does not function through a single dominant toxic pathway but through integrated behavioural and structural disruption. Such multi-target action may reduce selective pressure and provides a framework for developing botanical formulations aimed at population-level suppression. With respect to chemosensory disruption, the molecular docking prediction of myristicin binding to OBP3 is consistent with the strong oviposition deterrence observed, but definitive confirmation of olfactory interference requires functional validation. Necessary experiments include fluorescence competitive binding assays with recombinant AalbOBP3, electrophysiological recordings (single sensillum or electroantennography) using myristicin as a stimulus, dual-port olfactometer behavioural assays, and RNAi-mediated OBP3 knockdown followed by behavioural phenotyping. Until such studies are performed, the role of OBP3 in the observed deterrence remains an *in silico*-informed hypothesis. Future work should additionally prioritise formulation optimisation, field validation, residual-efficacy testing under environmental conditions, and integration into comprehensive vector management frameworks to determine practical applicability in real-world settings.

## 5 Conclusions

MFEO exhibited integrated disruption across multiple mosquito life stages, including oviposition avoidance, embryonic mortality, larval lethality, and adult knockdown. Molecular docking analysis further supported a mechanistic hypothesis of interaction with mosquito odorant-binding proteins, with myristicin exhibiting the strongest predicted binding affinity toward OBP3 (−6.8 kcal/mol). Molecular dynamics validation (RMSF < 2 Å) indicates conformational stability of the predicted complex under simulated conditions. Experimental validations, for example through fluorescence competitive binding assays or RNAi-mediated OBP knockdown, will be necessary to confirm functional disruption of olfactory signalling. The combined effects of behavioural modification (oviposition deterrence), structural damage to eggs, larval toxicity, and predicted interaction with olfactory proteins position MFEO as a promising candidate for integrated vector management strategies that emphasize behavioural disruption alongside mortality-based control.

## Ethical approval

Not applicable; this study did not involve vertebrate animals or human participants. All experiments were conducted on *Ae. albopictus* (Insecta: Diptera), an invertebrate species not covered by national or institutional regulations governing animal experimentation; ethical approval was therefore not required. Field collection of mosquito eggs was authorised by the National and Political Unity Agency of Central Java (Kesbangpol) under permit no. 070/642/XII/5.5/2021, and written consent was obtained from residents and landowners prior to ovitrap placement.

## CRediT authorship contribution statement

**Penny Humaidah Hamid:** Conceptualization, Methodology, Investigation, Formal analysis, Project administration, Writing - original draft, Writing - review & editing. **Arif Nur Muhammad Ansori:** Formal analysis, Investigation, Validation, Resources, Writing - review & editing. **Mochammad Aqilah Herdiansyah:** Investigation, Visualization, Validation, Writing - review & editing. **Herjuno Ari Nugroho:** Investigation, Resources, Writing - review & editing. **Ahmad Ghiffari:** Methodology, Investigation, Writing - review & editing. **Mujiyanto Mujiyanto:** Investigation, Resources, Writing - review & editing. **Kartika Dyah Palupi:** Investigation, Formal analysis, Resources, Writing - review & editing.

## Statement on the use of AI-assisted technologies

During the preparation of this work, the authors used ChatGPT to improve the English. After using this tool, the authors reviewed and edited the content as needed and take full responsibility for the content of the published article.

## Funding

Equipment, reagents and consumables were provided through E-Layanan Sains, Badan Riset dan Inovasi Nasional (BRIN), Indonesia. Grant support was provided by the Tropical Onehealth and Ecohealth Institute, Indonesia.

## Declaration of competing interests

The authors declare that they have no known competing financial interests or personal relationships that could have appeared to influence the work reported in this paper.

## Data Availability

The data supporting the conclusions of this article are included within the article and its supplementary file.

## References

[bib1] Abbas M.G., Azeem M., Bashir M.U., Ali F., Mozūraitis R., Binyameen M. (2024). Chemical composition, repellent, and oviposition deterrent potential of wild plant essential oils against three mosquito species. Molecules.

[bib2] Abourashed E.A., El-Alfy A.T. (2016). Chemical diversity and pharmacological significance of the secondary metabolites of nutmeg (*Myristica fragrans* Houtt.). Phytochem. Rev..

[bib3] Aini N.S., Ansori A.N.M., Herdiansyah M.A., Kharisma V.D., Widyananda M.H., Murtadlo A.A.A. (2024). Antimalarial potential of phytochemical compounds from *Garcinia atroviridis* Griff. ex T. Anders targeting multiple proteins of *Plasmodium falciparum* 3D7: an *in silico* approach. BIO Integr..

[bib4] Andrade-Ochoa S., Sánchez-Aldana D., Chacón-Vargas K.F., Rivera-Chavira B.E., Sánchez-Torres L.E., Camacho A.D. (2018). Oviposition deterrent and larvicidal and pupaecidal activity of seven essential oils and their major components against *Culex quinquefasciatus* Say (Diptera: Culicidae): synergism-antagonism effects. Insects.

[bib5] Ashokkumar K., Simal-Gandara J., Murugan M., Dhanya M.K., Pandian A. (2022). Nutmeg (*Myristica fragrans* Houtt.) essential oil: a review on its composition, biological, and pharmacological activities. Phytother. Res..

[bib6] Bae I.K., Kim K., Choi S.-D., Chang K.-S., Lee H.-S., Lee S.-E. (2017). Mosquito larvicidal activities of naturally occurring compounds derived from *Piper* species. Appl. Biol. Chem..

[bib7] Baker E.N., Hubbard R.E. (1984). Hydrogen bonding in globular proteins. Prog. Biophys. Mol. Biol..

[bib8] Benedict M.Q., Levine R.S., Hawley W.A., Lounibos L.P. (2007). Spread of the tiger: global risk of invasion by the mosquito *Aedes albopictus*. Vector Borne Zoonotic Dis..

[bib9] Bonizzoni M., Gasperi G., Chen X., James A.A. (2013). The invasive mosquito species *Aedes albopictus*: current knowledge and future perspectives. Trends Parasitol..

[bib10] Brixius D. (2018). A hard nut to crack: nutmeg cultivation and the application of natural history between the Maluku Islands and Isle de France (1750s-1780s). Br. J. Hist. Sci..

[bib11] Campbell B.E., Pereira R.M., Koehler P.G., Trdan S. (2016). Insecticides Resistance.

[bib12] Cossetin L.F., Santi E.M.T., Garlet Q.I., Matos A.F.I.M., De Souza T.P., Loebens L. (2021). Comparing the efficacy of nutmeg essential oil and a chemical pesticide against *Musca domestica* and *Chrysomya albiceps* for selecting a new insecticide agent against synantropic vectors. Exp. Parasitol..

[bib13] Dekel A., Sar-Shalom E., Vainer Y., Yakir E., Bohbot J.D. (2022). The ovipositor cue indole inhibits animal host attraction in *Aedes aegypti* (Diptera: Culicidae) mosquitoes. Parasites Vectors.

[bib14] Delatte H., Desvars A., Bouétard A., Bord S., Gimonneau G., Vourc’h G., Fontenille D. (2010). Blood-feeding behavior of *Aedes albopictus*, a vector of chikungunya on La Réunion. Vector Borne Zoonotic Dis..

[bib15] Deng Y., Yan H., Gu J., Xu J., Wu K., Tu Z., James A.A., Chen X. (2013). Molecular and functional characterization of odorant-binding protein genes in an invasive vector mosquito, *Aedes albopictus*. PLoS One.

[bib16] Farnesi L.C., Menna-Barreto R.F.S., Martins A.J., Valle D., Rezende G.L. (2015). Physical features and chitin content of eggs from the mosquito vectors *Aedes aegypti*, *Anopheles aquasalis* and *Culex quinquefasciatus*: connection with distinct levels of resistance to desiccation. J. Insect Physiol..

[bib17] Farnesi L.C., Vargas H.C.M., Valle D., Rezende G.L. (2017). Darker eggs of mosquitoes resist more to dry conditions: melanin enhances serosal cuticle contribution in egg resistance to desiccation in *Aedes*, *Anopheles* and *Culex* vectors. PLoS Negl. Trop. Dis..

[bib18] Feng Y.-X., Wang Y., You C.-X., Guo S.-S., Du Y.-S., Du S.-S. (2019). Bioactivities of patchoulol and phloroacetophenone from *Pogostemon cablin* essential oil against three insects. Int. J. Food Prop..

[bib19] Gayathiri E., Prakash P., Kumaravel P., Jayaprakash J., Ragunathan M.G., Sankar S. (2023). Computational approaches for modeling and structural design of biological systems: a comprehensive review. Prog. Biophys. Mol. Biol..

[bib20] Gloria-Soria A., Payne A.F., Bialosuknia S.M., Stout J., Mathias N., Eastwood G. (2020). Vector competence of *Aedes albopictus* populations from the northeastern United States for chikungunya, dengue, and Zika viruses. Am. J. Trop. Med. Hyg..

[bib21] Govindarajan M., Rajeswary M., Hoti S.L., Benelli G. (2016). Larvicidal potential of carvacrol and terpinen-4-ol from the essential oil of *Origanum vulgare* (Lamiaceae) against *Anopheles stephensi*, *Anopheles subpictus*, *Culex quinquefasciatus* and *Culex tritaeniorhynchus* (Diptera: Culicidae). Res. Vet. Sci..

[bib22] Hallem E.A., Carlson J.R. (2006). Coding of odors by a receptor repertoire. Cell.

[bib24] Hamid P.H., Ninditya V.I., Ghiffari A., Taubert A., Hermosilla C. (2020). The V1016G mutation of the voltage-gated sodium channel (VGSC) gene contributes to the insecticide resistance of *Aedes aegypti* from Makassar, Indonesia. Parasitol. Res..

[bib23] Hamid P.H., Ninditya V.I., Prastowo J., Haryanto A., Taubert A., Hermosilla C. (2018). Current status of *Aedes aegypti* insecticide resistance development from Banjarmasin, Kalimantan, Indonesia. Biomed Res. Int..

[bib25] Herdiansyah M.A., Rizaldy R., Alifiansyah M.R., Fetty A.J., Anggraini D., Agustina N. (2024). Molecular interaction analysis of ferulic acid (4-hydroxy-3-methoxycinnamic acid) as main bioactive compound from palm oil waste against MCF-7 receptors: an *in silico* study. Narrat. J..

[bib26] Hummelbrunner L.A., Isman M.B. (2001). Acute, sublethal, antifeedant, and synergistic effects of monoterpenoid essential oil compounds on the tobacco cutworm, *Spodoptera litura* (Lep., Noctuidae). J. Agric. Food Chem..

[bib27] Jung W.-C., Jang Y.-S., Hieu T.T., Lee C.-K., Ahn Y.-J. (2007). Toxicity of *Myristica fragrans* seed compounds against *Blattella germanica* (Dictyoptera: Blattellidae). J. Med. Entomol..

[bib28] Kamal M., Kenawy M.A., Rady M.H., Khaled A.S., Samy A.M. (2018). Mapping the global potential distributions of two arboviral vectors *Aedes aegypti* and *Ae. albopictus* under changing climate. PLoS One.

[bib29] Khaleel C., Tabanca N., Buchbauer G. (2018). α-Terpineol, a natural monoterpene: a review of its biological properties. Open Chem..

[bib30] Kingsolver J.G., Woods H.A., Buckley L.B., Potter K.A., MacLean H.J., Higgins J.K. (2011). Complex life cycles and the responses of insects to climate change. Integr. Comp. Biol..

[bib32] Kraemer M.U.G., Reiner R.C., Brady O.J., Messina J.P., Gilbert M., Pigott D.M. (2019). Past and future spread of the arbovirus vectors *Aedes aegypti* and *Aedes albopictus*. Nat. Microbiol..

[bib31] Kraemer M.U.G., Sinka M.E., Duda K.A., Mylne A.Q.N., Shearer F.M., Barker C.M. (2015). The global distribution of the arbovirus vectors *Aedes aegypti* and *Ae. albopictus*. eLife.

[bib33] Li M., Liu S., Yin Z., Bernigaud C., Guillot J., Fang F. (2021). Activity of terpenes derived from essential oils against *Sarcoptes scabiei* eggs. Parasites Vectors.

[bib34] Mao W., Zangerl A.R., Berenbaum M.R., Schuler M.A. (2008). Metabolism of myristicin by *Depressaria pastinacella* CYP6AB3v2 and inhibition by its metabolite. Insect Biochem. Mol. Biol..

[bib35] Martianasari R., Hamid P.H. (2019). Larvicidal, adulticidal, and oviposition-deterrent activity of *Piper betle* L. essential oil to *Aedes aegypti*. Vet. World.

[bib36] Martínez-Mercado J.P., Sierra-Santoyo A., Verdín-Betancourt F.A., Rojas-García A.E., Quintanilla-Vega B. (2022). Temephos, an organophosphate larvicide for residential use: a review of its toxicity. Crit. Rev. Toxicol..

[bib37] MoARI (2021). [Regulation of the Minister of Agriculture of the Republic of Indonesia No. 19 of 2021 on plant genetic resources and the release of plantation crop varieties]. Ministry of Agriculture of the Republic of Indonesia, Jakarta.

[bib38] Moungthipmalai T., Puwanard C., Aungtikun J., Sittichok S., Soonwera M. (2023). Ovicidal toxicity of plant essential oils and their major constituents against two mosquito vectors and their non-target aquatic predators. Sci. Rep..

[bib39] Mundim-Pombo A.P.M., Carvalho H.J.C., Rodrigues Ribeiro R., León M., Maria D.A., Miglino M.A. (2021). *Aedes aegypti*: egg morphology and embryonic development. Parasites Vectors.

[bib40] Norris E.J., Archevald-Cansobre M., Gross A.D., Bartholomay L.C., Coats J.R. (2018). Rapid immobilization of adult *Aedes aegypti* caused by plant essential oils at sublethal concentrations. J. Am. Mosq. Control Assoc..

[bib41] Park Y.-L., Tak J.H., Preedy V.R. (2016). Essential Oils in Food Preservation, Flavor and Safety.

[bib42] Passara H., Sittichok S., Sinthusiri J., Moungthipmalai T., Puwanard C., Murata K., Soonwera M. (2024). Ovicidal toxicity and morphological changes in housefly eggs induced by the essential oils of star anise and lemongrass and their main constituents. Insects.

[bib43] Pereira-dos-Santos T., Roiz D., Lourenço-de-Oliveira R., Paupy C. (2020). A systematic review: is *Aedes albopictus* an efficient bridge vector for zoonotic arboviruses?. Pathogens.

[bib44] Phasomkusolsil S., Soonwera M. (2012). The effects of herbal essential oils on the oviposition-deterrent and ovicidal activities of *Aedes aegypti* (Linn.), *Anopheles dirus* (Peyton & Harrison) and *Culex quinquefasciatus* (Say). Trop. Biomed..

[bib45] Portilla-Pulido J.S., Castillo-Morales R.M., Barón-Rodríguez M.A., Duque J.E., Méndez-Sánchez S.C. (2020). Design of a repellent against *Aedes aegypti* (Diptera: Culicidae) using *in silico* simulations with AaegOBP1 protein. J. Med. Entomol..

[bib46] Reinhold J.M., Lazzari C.R., Lahondère C. (2018). Effects of the environmental temperature on *Aedes aegypti* and *Aedes albopictus* mosquitoes: a review. Insects.

[bib47] Rezende G.L., Martins A.J., Gentile C., Farnesi L.C., Pelajo-Machado M., Peixoto A.A., Valle D. (2008). Embryonic desiccation resistance in *Aedes aegypti*: presumptive role of the chitinized serosal cuticle. BMC Dev. Biol..

[bib48] Scalerandi E., Flores G.A., Palacio M., Defagó M.T., Carpinella M.C., Valladares G. (2018). Understanding synergistic toxicity of terpenes as insecticides: contribution of metabolic detoxification in *Musca domestica*. Front. Plant Sci..

[bib49] Seneme E.F., dos Santos D.C., Silva E.M.R., Franco Y.E.M., Longato G.B. (2021). Pharmacological and therapeutic potential of myristicin: a literature review. Molecules.

[bib50] Silva J.R.A., Oliveira A.A., França L.P., da Cruz J.D., Amaral A.C.F. (2024). Exploring the larvicidal and adulticidal activity against *Aedes aegypti* of essential oil from *Bocageopsis multiflora*. Molecules.

[bib51] Songtawee N., Gleeson M.P., Choowongkomon K. (2013). Computational study of EGFR inhibition: molecular dynamics studies on the active and inactive protein conformations. J. Mol. Model..

[bib52] Soonwera M., Sittichok S. (2020). Adulticidal activities of *Cymbopogon citratus* (Stapf.) and *Eucalyptus globulus* (Labill.) essential oils and of their synergistic combinations against *Aedes aegypti* (L.), *Aedes albopictus* (Skuse), and *Musca domestica* (L.). Environ. Sci. Pollut. Res..

[bib53] Spinozzi E., Maggi F., Bonacucina G., Pavela R., Boukouvala M.C., Kavallieratos N.G. (2021). Apiaceae essential oils and their constituents as insecticides against mosquitoes - a review. Ind. Crops Prod..

[bib54] Van Gils C., Cox P.A. (1994). Ethnobotany of nutmeg in the Spice Islands. J. Ethnopharmacol..

[bib55] Wheelwright M., Whittle C.R., Riabinina O. (2021). Olfactory systems across mosquito species. Cell Tissue Res..

[bib56] WHO (2016).

[bib57] Yan R., Xu Z., Qian J., Zhou Q., Wu H., Liu Y. (2022). Molecular and functional characterization of a conserved odorant receptor from *Aedes albopictus*. Parasites Vectors.

[bib58] Yang C., Chen E.A., Zhang Y. (2022). Protein-ligand docking in the machine-learning era. Molecules.

[bib59] Yang Y.C., Lee S.H., Clark J.M., Ahn Y.J. (2009). Ovicidal and adulticidal activities of *Origanum majorana* essential oil constituents against insecticide-susceptible and pyrethroid/malathion-resistant *Pediculus humanus capitis* (Anoplura: Pediculidae). J. Agric. Food Chem..

[bib60] Yongsue T., Tainchum K. (2025). Establishing discriminating concentrations of indigenous plant-based insecticides against adult *Aedes aegypti* (L.) and *Aedes albopictus* Skuse (Diptera: Culicidae). J. Vector Ecol..

