# Supplementary material for: Multi-stage insecticide potentials of Myristica fragrans against the invasive Aedes albopictus, and putative binding mechanism of myristicin targeting the odorant-binding proteins (OBPs)
Source: Curr Res Parasitol Vector Borne Dis. 2026 Aug 7;10:100424. doi: 10.1016/j.crpvbd.2026.100424 (PMC13487829; doi:10.1016/j.crpvbd.2026.100424)
Supplement: Supplementary file 1 [file mmc1.pdf]

Supplementary materials

Supplementary Table S1. Annotated odorant-binding proteins (OBPs) of *Aedes albopictus* with transcript- or protein-level evidence, used as docking targets for myristicin in this study. For each OBP, the table reports the standardised OBP designation, functional annotation (where available in the source databases), and accession identifiers from the NCBI Reference Sequence (RefSeq) and UniProt/protein RefSeq databases.

General name	Description	NCBI Reference Sequence	UniProt/ protein refseq NCBI
**obp1**	odorant-binding protein 1 [*Aedes albopictus* (Asian tiger mosquito) (*Stegomyia albopicta*)]	JN874410.1	H9A9Y0
**obp3**	odorant-binding protein 3 [*Aedes albopictus* (Asian tiger mosquito) (*Stegomyia albopicta*)]	JN874412.1	H9A9Y2
**obp5**	general odorant-binding protein 67 [*Aedes albopictus* (Asian tiger mosquito)]	NC_085137.1	M4VNZ9
**obp6**	general odorant-binding protein 56d [*Aedes albopictus*]	NC_085138.1	K0A2J7
**obp10**	obp10 [*Aedes albopictus*]	KC405540.1	AGI04311.1
**obp11**	general odorant-binding protein 72 [*Aedes albopictus*]	XP_019562477.1	M4W725
**obp13**	general odorant-binding protein 56a [*Aedes albopictus*]	XP_019531746.3	M4VRD7
**obp46**	atypical odorant-binding protein 46	FJ040863.1	B5TYS7
**obp19**	uncharacterized LOC134291994 [*Aedes albopictus* (Asian tiger mosquito)]	NC_085138.1	XP_062716570.1
**obp20**	odorant-binding protein 84a [*Aedes albopictus* (Asian tiger mosquito)]	NC_085138.1	XP_019565322.3
**obp21**	general odorant-binding protein 99a [*Aedes albopictus* (Asian tiger mosquito)]	NC_085136.1	XP_029717520.2
**obp24**	uncharacterized LOC109401575 [*Aedes albopictus* (Asian tiger mosquito)]	NC_085138.1	M4VSM7
**obp37**	obp37 [*Aedes albopictus*]	KC405549	M4VP05
**obp38**	pheromone-binding protein-related protein 6 [*Aedes albopictus* (Asian tiger mosquito)]	NC_085136.1	M4VPE8
**obp39**	general odorant-binding protein 83a [*Aedes albopictus* (Asian tiger mosquito)]	NC_085136.1	M4W733
**obp42**	general odorant-binding protein 67 [*Aedes albopictus* (Asian tiger mosquito)	NC_085137.1	A0A1W7R7G5
**obp55**	general odorant-binding protein 72 [*Aedes albopictus*]	XP_019562477.1	M4VP10
**obp56**	general odorant-binding protein 83a [*Aedes albopictus*]	XP_029736214.1	H9A9Y1
**obp59**	obp59 [*Aedes albopictus*]	KC405556.1	M4W737
**obp62**	obp62 [*Aedes albopictus*]	KC405558.1	AGI04329.1
**obp67**	atypical odorant-binding protein 67	FJ040862.1	B5TYS6
**obp68**	atypical odorant-binding protein 68	FJ040861.1	B5TYS5

Supplementary Table S2. Neurophysiological target proteins of *Aedes albopictus* selected for comparative molecular docking analysis with myristicin and the reference synthetic repellent N,N-diethyl-3-methylbenzamide.

General name	Description	NCBI reference sequence	UniProt/protein RefSeq NCBI
**AChE**	Acetylcholinesterase isoform X1 [*Aedes albopictus* Asian tiger mosquito]	XM_029857745.1	translated protein model from XM_029857745.1
**AChE-like**	Acetylcholinesterase-like / butyrylcholinesterase-like protein [*Aedes albopictus*]	XP_019540308.1	XP_019540308.1
**GABA-B-R2**	Gamma-aminobutyric acid type B receptor subunit 2 [*Aedes albopictus*]	XP_019540919.1	XP_019540919.2
**GABA-A alpha-6**	Gamma-aminobutyric acid type A receptor subunit alpha-6 [*Aedes albopictus*]	XP_019547850.1	LOC109428426
**VGSC / para**	Voltage-gated sodium channel para [*Aedes albopictus*]	NP_001393189.1	NP_001393189.1
**Octβ2R**	Octopamine receptor beta-2R [*Aedes albopictus*]	XP_062716640.1	XP_062716640.1
**Octβ2R-like**	Octopamine receptor beta-2R-like [*Aedes albopictus*]	XP_019547601.1	XP_019547601.1

Supplementary Table S3. Neurophysiological target proteins of *Aedes albopictus* selected for comparative molecular docking analysis with myristicin and the reference synthetic repellent N,N-diethyl-3-methylbenzamide.

General name	Description	NCBI reference sequence	UniProt/protein RefSeq NCBI
**AChE**	Acetylcholinesterase isoform X1 [*Aedes albopictus* Asian tiger mosquito]	XM_029857745.1	translated protein model from XM_029857745.1
**AChE-like**	Acetylcholinesterase-like/ butyrylcholinesterase-like protein [*Aedes albopictus*]	XP_019540308.1	XP_019540308.1
**GABA-B-R2**	Gamma-aminobutyric acid type B receptor subunit 2 [*Aedes albopictus*]	XP_019540919.1	XP_019540919.2
**GABA-A alpha-6**	Gamma-aminobutyric acid type A receptor subunit alpha-6 [*Aedes albopictus*]	XP_019547850.1	LOC109428426
**VGSC/para**	Voltage-gated sodium channel para [*Aedes albopictus*]	NP_001393189.1	NP_001393189.1
**Octβ2R**	Octopamine receptor beta-2R [*Aedes albopictus*]	XP_062716640.1	XP_062716640.1
**Octβ2R-like**	Octopamine receptor beta-2R-like [*Aedes albopictus*]	XP_019547601.1	XP_019547601.1

Supplementary Table S4. Predicted binding affinities and residue-level interaction profiles of myristicin with selected neurophysiological target proteins of *Aedes albopictus*, including acetylcholinesterase (AChE) isoforms, gamma-aminobutyric acid (GABA) receptor subunits, voltage-gated sodium channel (VGSC), and octopamine receptor (OR) isoforms. Only the most stable binding conformation is reported for each receptor.

Complex ligand-receptor	Complex binding affinity (kcal/mol)	Type of interaction	Amino acids involved
Myristicin-AChE	-6.3	vdW	Phe416(A), Phe457(A), Cys414(A), Ile198(A), Asp200(A)
		HI	Tyr461(A), Ile413(A)
		PHI	Tyr456(A)
Myristicin-AChE-like	-4.7	vdW	Leu496(A), Ser27(A), Val499(A), Tyr504(A), Asn25(A)
		HI	Tyr503(A)
		PHI	Glu500(A)
Myristicin-GABA-B-R2	-6.0	vdW	Gly706(B), Ser744(B)
		HI	Lys705(B), Phe602(B), Leu709(B), Ala745(B)
		PHI	-
Myristicin-GABA-A alpha-6	-5.6	vdW	Glu308(A), Ala283(A), His306(A), Trp309(A), Thr459(A), Gln451(A)
		HI	Phe346(A)
		PHI	Ser455(A)
Myristicin-VGSC/para	-5.3	vdW	His196(A), Ile197(A), Ile200(A), Gln171(A)
		HI	Leu(A), Leu189(A), Ala193(A), Leu167(A), Lys168(A)
		PHI	Thr164(A)
Myristicin-Octβ2R	-4.9	vdW	Asn478(A), Tyr474(A), Phe477(A), Phe481(A)
		HI	Leu422(A), Arg420(A), Ile425(A), Ile424(A), Ile473(A)
		PHI	-
Myristicin-Octβ2R-like	-5.6	vdW	Ser1076(A), Gly1074(A), Gly1019(A)
		HI	His930(A), Trp1096(A)

Abbreviations: vdW, van der Waals interaction; HI, hydrophobic interaction; PHI, polar hydrogen interaction.

Supplementary Table S5. Predicted binding affinities and residue-level interaction profiles of N,N-diethyl-3-methylbenzamide (DEET) to 19 annotated odorant-binding proteins (OBPs) of *Aedes albopictus*. Binding energies (kcal/mol) indicate the strength of ligand-receptor interactions, while interacting amino acid residues are categorized based on van der Waals (vdW), hydrophobic interactions (HI), and polar hydrogen interactions (PHI).

Complex ligand-receptor	Complex binding affinity (kcal/mol)	Type of interaction	Amino acids involved
DEET-OBP1	-7.0	vdW	Trp50(A), Phe52(A), Leu87(A), Gln86(A), Ser134(A), Ile135(A), Met69(A)
		HI	Tyr61(A), Arg30(A), Leu26(A), Ile62(A), Val65(A), Ile131(A), Phe66(A), Leu71(A)
		PHI	-
DEET-OBP3	-6.6	vdW	Val65(A), Gln64(A), Leu81(A), Ser61(A), Phe77(A)
		HI	Leu102(A), Leu44(A), Leu114(A), Ile41(A), Leu101(A), Met48(A), Val50(A)
		PHI	-
DEET-OBP5	-5.1	vdW	Gln163(A), Gln167(A), Ala15(A), Gln18(A), Lys183(A), Ser19(A)
		HI	Tyr22(A)
		PHI	-
DEET-OBP6	-5.1	vdW	Val40(A), Thr44(A), Glu52(A), Gly55(A), Ala54(A), Asp57(A)
		HI	Ala41(A), Leu48(A), Ala56(A)
		PHI	Thr60(A), Lys47(A)
DEET-OBP10	-4.6	vdW	Leu81(A), Glu33(A), Arg80(A), Tyr37(A), Ser95(A)
		HI	Leu99(A), Lys92(A), Leu79(A)
		PHI	-
DEET-OBP11	-6.6	vdW	Asp127(A), Gln23(A), Thr126(A), Thr24(A), Gln56(A)
		HI	Leu55(A)
		PHI	-
DEET-OBP13	-6.4	vdW	Phe69(A), Lys89(A), Val103(A), Cys106(A)
		HI	Leu90(A), Val102(A), Met81(A), Leu121(A), Val74(A), Val86(A)
		PHI	-
DEET-OBP46	-5.0	vdW	Pro272(A), Asp135(A), His59(A), Thr64(A), Asn62(A), Thr134(A), Asp63(A)
		HI	Val56(A), Trp61(A), Tyr273(A)
		PHI	-
DEET-OBP24	-6.8	vdW	Val72(A), Leu78(A), Ser154(A), Leu81(A)
		HI	Trp192(A), Tyr155(A), Val34(A), Phe151(A), Val82(A)
		PHI	-
DEET-OBP37	-4.6	vdW	Asp67(A)
		HI	Lys140(A), Pro143(A)
		PHI	Gly66(A)
DEET-OBP38	-5.2	vdW	Glu18(A), Cys16(A), Gln58(A), Glu59(A), Glu131(A)
		HI	Val60(A), Lys130(A), Pro134(A), Pro19(A)
		PHI	Gln17(A), Asp57(A)
DEET-OBP39	-7.3	vdW	Gly110(A), Phe33(A), Tyr140(A), His139(A), Tyr28(A), Phe77(A)

**Complex ligand-receptor**	**Complex binding affinity (kcal/mol)**	**Type of interaction**	**Amino acids involved**
		HI	His95(A), Leu94(A), Met102(A), Phe141(A), Leu98(A)
		PHI	-
DEET-OBP42	-5.5	vdW	Leu158(A), Met161(A), Pro43(A), Thr42(A), Met193(A), Gly162(A)
		HI	Leu165(A), Met44(A), Met51(A)
		PHI	Met46(A), Leu45(A)
DEET-OBP55	-6.4	vdW	Ile101(A), Val87(A), Gln100(A), Ile104(A), Pro151(A)
		HI	Leu92(A), Val86(A), Ile137(A), Leu117(A), Ala97(A), Met84(A), Val141(A), Met105(A)
		PHI	-
DEET-OBP56	-6.4	vdW	Leu142(A), Ile143(A), Leu37(A), Ala106(A), Leu98(A), Leu94(A)
		HI	Tyr140(A), Met109(A), His129(A), Phe77(A), Leu76(A)
		PHI	-
DEET-OBP59	-5.8	vdW	Tyr60(A), Phe94(A), Ile95(A), Leu77(A), Asn78(A)
		HI	Trp121(A)
		PHI	-
DEET-OBP62	-5.7	vdW	Arg159(A), Ile193(A), Gly74(A), Arg73(A)
		HI	Pro72(A), Pro152(A), Ile133(A), Ile134(A), Ala156(A)
		PHI	Cys75(A), Gly155(A)
DEET-OBP67	-5.2	vdW	Thr141(A), Phe253(A), Glu146(A)
		HI	Tyr325(A), Val138(A), Met145(A), Arg142(A)
		PHI	-
DEET-OBP68	-5.1	vdW	Gly76(A), Gly204(A), Tyr205(A), Tyr88(A), Thr92(A)

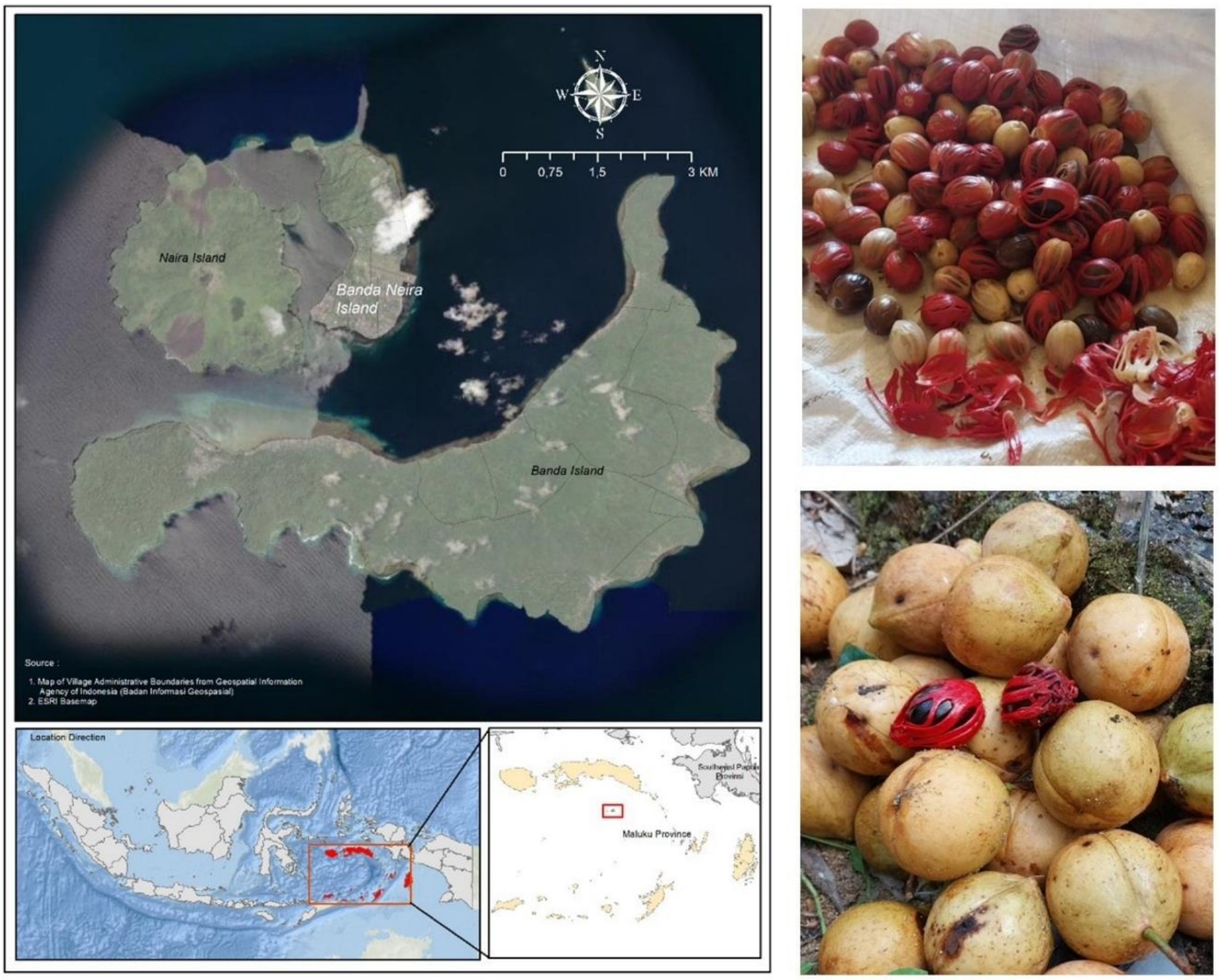

Supplementary Figure S1. Geographical location of *Myristica fragrans* collection site in the Banda Islands, Maluku Province, Indonesia. The map indicates the specific sampling area, with regional and national location references. *Myristica fragrans* produces a fleshy drupe-like fruit that splits open upon maturity. The outer yellow pericarp (husk) encloses a hard seed (nutmeg), which is covered by a bright red aril known as mace. The seed consists of a woody seed coat (testa) surrounding the kernel (endosperm), which contains the essential oil. Photographs show harvested nutmeg fruits and seeds used for essential oil extraction.

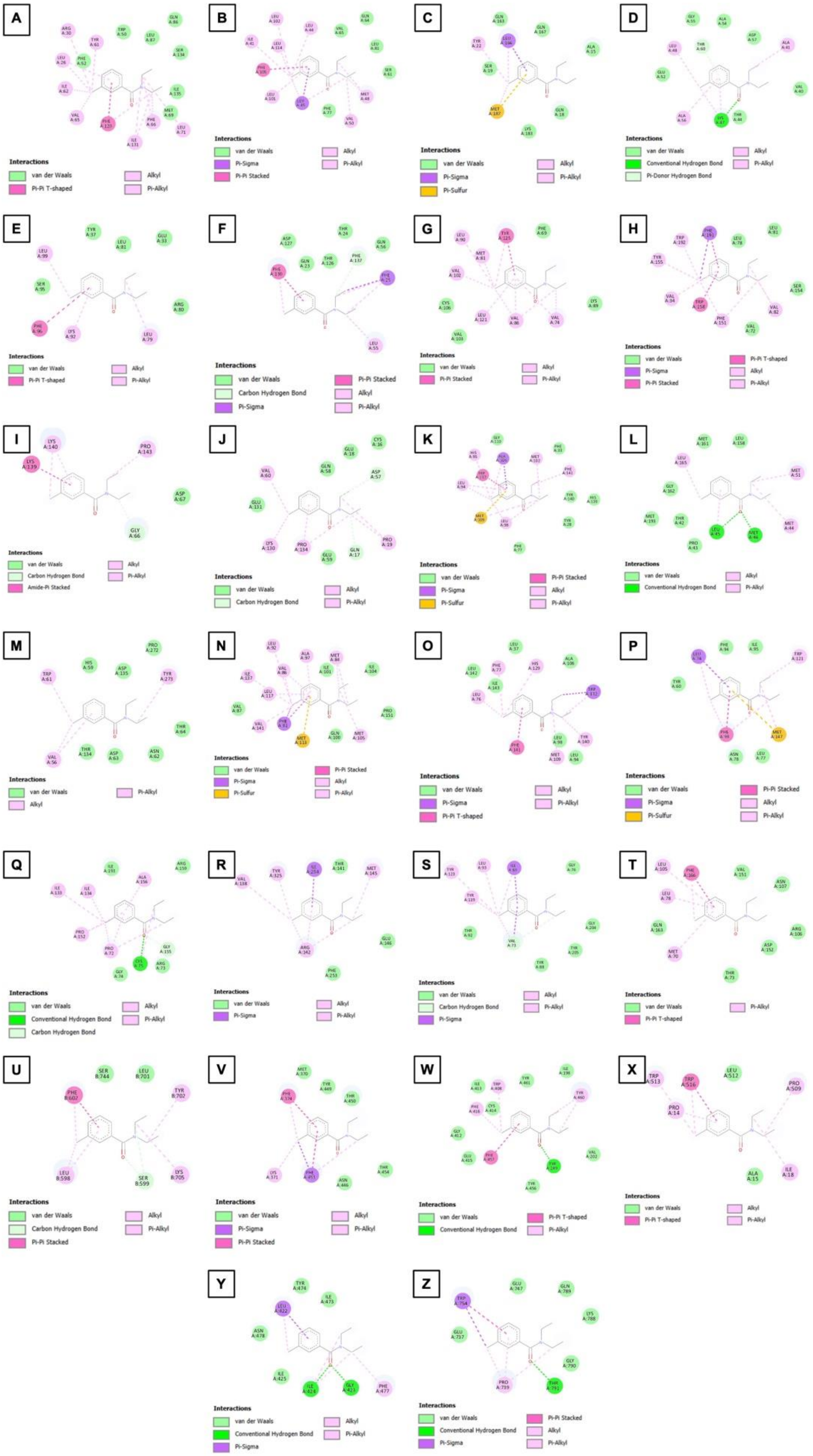

A
Interactions
van der Waals
Pi-Pi T-shaped
Alkyl
Pi-Alkyl
B
Interactions
van der Waals
Pi-Sigma
Pi-Pi Stacked
Alkyl
Pi-Alkyl
C
Interactions
van der Waals
Pi-Sigma
Pi-Sulfur
Alkyl
Pi-Alkyl
D
Interactions
van der Waals
Conventional Hydrogen Bond
Pi-Donor Hydrogen Bond
Alkyl
Pi-Alkyl
E
Interactions
van der Waals
Pi-Pi T-shaped
Alkyl
Pi-Alkyl
F
Interactions
van der Waals
Carbon Hydrogen Bond
Pi-Sigma
Pi-Pi Stacked
Alkyl
Pi-Alkyl
G
Interactions
van der Waals
Pi-Pi Stacked
Alkyl
Pi-Alkyl
H
Interactions
van der Waals
Pi-Sigma
Pi-Pi Stacked
Pi-Pi T-shaped
Alkyl
Pi-Alkyl
I
Interactions
van der Waals
Carbon Hydrogen Bond
Amide-Pi Stacked
Alkyl
Pi-Alkyl
J
Interactions
van der Waals
Carbon Hydrogen Bond
Alkyl
Pi-Alkyl
K
Interactions
van der Waals
Pi-Sigma
Pi-Sulfur
Pi-Pi Stacked
Alkyl
Pi-Alkyl
L
Interactions
van der Waals
Conventional Hydrogen Bond
Alkyl
Pi-Alkyl
M
Interactions
van der Waals
Alkyl
Pi-Alkyl
N
Interactions
van der Waals
Pi-Sigma
Pi-Sulfur
Pi-Pi Stacked
Alkyl
Pi-Alkyl
O
Interactions
van der Waals
Pi-Sigma
Pi-Pi T-shaped
Alkyl
Pi-Alkyl
P
Interactions
van der Waals
Pi-Sigma
Pi-Sulfur
Pi-Pi Stacked
Alkyl
Pi-Alkyl
Q
Interactions
van der Waals
Conventional Hydrogen Bond
Carbon Hydrogen Bond
Alkyl
Pi-Alkyl
R
Interactions
van der Waals
Pi-Sigma
Alkyl
Pi-Alkyl
S
Interactions
van der Waals
Carbon Hydrogen Bond
Pi-Sigma
Alkyl
Pi-Alkyl
T
Interactions
van der Waals
Pi-Pi T-shaped
Pi-Alkyl
U
Interactions
van der Waals
Carbon Hydrogen Bond
Pi-Pi Stacked
Alkyl
Pi-Alkyl
V
Interactions
van der Waals
Pi-Sigma
Pi-Pi Stacked
Alkyl
Pi-Alkyl
W
Interactions
van der Waals
Conventional Hydrogen Bond
Pi-Pi T-shaped
Pi-Alkyl
X
Interactions
van der Waals
Pi-Pi T-shaped
Alkyl
Pi-Alkyl
Y
Interactions
van der Waals
Conventional Hydrogen Bond
Pi-Sigma
Alkyl
Pi-Alkyl
Z
Interactions
van der Waals
Conventional Hydrogen Bond
Pi-Sigma
Pi-Pi Stacked
Alkyl
Pi-Alkyl

Supplementary Figure S2. Two-dimensional interaction diagrams of predicted molecular docking between the synthetic repellent N,N-diethyl-3-methylbenzamide (DEET) and *Aedes albopictus*: (A-S) 19 odorant-binding proteins (OBPs), (T) voltage-gated sodium channel (VGSC/para), (U-V) GABA receptor subunits (GABA-B-R2 and GABA-A α-6), (W-X) cholinesterase isoforms (AChE and AChE-like), and (Y-Z) octopamine receptor isoforms (Octβ2R and Octβ2R-like). DEET exhibited the strongest predicted binding affinities toward OBP39 (-7.3 kcal/mol), OBP1 (-7.0 kcal/mol), and OBP24 (-6.8 kcal/mol), surpassing myristicin's best OBP affinity (-6.8 kcal/mol for OBP3). However, DEET showed broader but generally weaker or comparable binding to VGSC (-5.7 kcal/mol), GABA-A α-6 (-6.5 kcal/mol), and AChE (-6.1 kcal/mol) relative to myristicin. These comparative *in silico* profiles highlight that while DEET preferentially targets OBPs with high affinity, myristicin displays a more balanced multi-receptor interaction spectrum, potentially underpinning its integrated ovicidal, larvicidal, adulticidal, and oviposition-deterrent activities.
